# Impact of Multi-Cancer Early Detection Test on Late-Stage Cancer Diagnosis

**DOI:** 10.1056/NEJMoa2505723

**Published:** 2026-09-22

**Authors:** Peter Sasieni, Peter Johnson, Thomas Round, Jane Warwick, Helen Jones, Saoirse Dolly, Lennard Lee, Yujin Lee, Sir Harpal Kumar, Wei Liang, Rebecca Smittenaar, Richard D. Neal, Charles Swanton

**Affiliations:** 1 Queen Mary University of London, London, UK; 2 NHS England, London, UK; 3 King’s College London, London, UK; 4 EMS Healthcare, Cheshire, UK; 5 Guy’s and St. Thomas’ NHS Foundation Trust, London, UK; 6 University of Oxford, Oxford, UK; 7 GRAIL Bio UK, Ltd., London, UK; 8 University of Exeter, Exeter, UK; 9 University College London, London, UK; 10 The Francis Crick Institute, London, UK

## Abstract

**Background:**

NHS-Galleri, a randomized controlled trial, evaluates the clinical utility of a blood-based multi-cancer early detection (MCED) test added to usual care among individuals aged 50-77 years in England.

**Methods:**

Peripheral blood was collected at up to 3 annual visits. Following baseline blood collection, participants were randomized 1:1 to intervention (blood tested) or control (blood stored) arms. Participants and study personnel were initially blinded. Reductions of stage III/IV (primary endpoint) and IV (key secondary) cancers of 12 prespecified types in intervention vs control were measured ~3 years after the last participant’s randomization.

**Results:**

Overall, 71,122 and 71,128 participants were randomized to intervention and control arm, respectively. The primary endpoint was not met (incidence rate ratio [IRR; intervention vs control]:1.03 [0.92-1.14], p=0.63). For secondary endpoints, the IRR for stage IV cancer after 3 screening rounds was 0.86 (0.74-1.00). <1% of participants experienced related adverse events, none were serious.

**Conclusions:**

In NHS-Galleri, we did not observe a reduction in stage III/IV cancers across 12 prespecified cancers after 3 screening rounds when MCED testing was added to standard-of-care. Further follow-up is planned to assess how findings for stage III/IV cancers and the difference in stage IV cancers evolve over time. (Funded by GRAIL, Inc.; ClinicalTrials.gov #NCT05611632)

## Background

The survival advantage of earlier-stage cancer detection reflects broader eligibility for curative treatment and lower disease burden.^1^ Standard-of-care screening contributes to earlier diagnosis and improved survival,^2^ yet recommended single-cancer screening accounts for <7% of all cancer diagnoses in England (US: <15%).^2,3^

Multi-cancer early detection (MCED) tests offer a different approach by simultaneously screening for multiple cancer types using a biological specimen (e.g., blood).^4–6^ One MCED test analyses methylation patterns in cell-free DNA (cfDNA)^6^ to detect a cancer signal and predict the associated tissue type or organ (cancer signal origin) has demonstrated detection across a broad range of cancer types. The test has shown strong performance in case-control^7^ and prospective clinical studies,^8,9^ as well as in real-world experience.^10–12^

Here, we report the primary results from the NHS-Galleri trial (NCT05611632), a randomized controlled trial evaluating the clinical utility of a clinically validated MCED test when added to usual care in a national healthcare system among participants aged 50-77 years at invitation, over 3 annual screening rounds.

## Methods

### Trial oversight and design

The NHS-Galleri trial (ISRCTN91431511, registration date: 16 September 2021; NCT05611632, registration date: 28 October 2022), sponsored and funded by GRAIL, Inc. (Menlo Park, CA, USA), is a prospective randomized controlled trial to evaluate the clinical utility and effectiveness of an MCED blood test (Galleri^®^ [GRAIL, Inc.]) when added to usual care.^6^ Briefly, eligible individuals (those aged 50-77 years who did not have a cancer diagnosis nor treatment within the 3 years before enrolment, and were not being investigated or under clinical suspicion for cancer; **Supplementary Methods section 1**) in 8 participating English Cancer Alliance regions were invited. Invitations, information sheets, and consent forms were developed with input from diverse members of the public.^13^ Further information on trial oversight and author responsibilities are provided in **Supplementary Methods section 2**. Study design details (e.g., recruitment methods, trial objectives) are provided in publications^6,13^ and **Supplementary Methods sections 1-11**.

GRAIL, collaborating with The Cancer Research UK Cancer Prevention Trials Unit, was responsible for study design, conduct, reporting, and data analysis. The trial protocol, available at nejm.org, (**Supplementary Methods section 10**) received national ethical approval from Wales Research Ethics Committee 1 (Ref: 21/WA/0141). An Independent Data Monitoring Committee (IDMC) oversaw and assessed clinical study progress, reviewed clinical/safety data, and provided design and conduct recommendations to GRAIL.

### Procedures

At initial appointments conducted in mobile phlebotomy clinics, after informed consent, a baseline peripheral blood sample was collected by routine venipuncture, and participants were then randomized 1:1 into intervention or control arm (Figure S1).^6^ All participants, except those for whom the study team had received death notification, were invited to 2 further annual (12 months±6 weeks) blood draws; participants missing the second visit could return for the third. All participants and trial staff were initially blinded; intervention-arm participants with a positive (cancer signal detected) MCED test result had results communicated by unblinded trial nurses.^13^ Unblinded statisticians reviewed safety data and presented results to the IDMC in closed biannual meetings.

Blood samples from intervention-arm participants were analyzed using the MCED test. The study team facilitated referral of participants with a positive result for diagnostic testing via established National Health Service (NHS) urgent cancer referral pathways, following existing anatomical site-specific suspected cancer guidelines based on the MCED test cancer signal origin prediction.^6,14^ All control-arm and remaining intervention-arm samples were stored for consented future testing.

Cancer outcomes were collected via linked national healthcare datasets. Participants were passively followed for cancer diagnoses and death, until emigration or consent withdrawal.

Important protocol deviations are summarized in Tables S1-S2.

### Statistical analysis

The statistical analysis plan is available in **Supplementary Methods section 11**. Microsimulation indicated ~140,000 participants were needed for ≥90% power for the between-arm primary endpoint after three screening rounds, with follow-up through 12 months after the last participant’s third visit, across varied natural history assumptions;^15^ an approximately 20% relative reduction in stage III/IV cancers at the 12 prespecified sites was projected under the then most likely scenario.^6^ All analyses comparing the two arms were intention-to-treat analyses (conducted among randomized participants [Randomized Analysis Set]).

Cancer diagnoses from randomization to 12 July 2025 registered by the National Cancer Registration and Analysis Service (NCRAS) were included, for a follow-up period of 3 screening rounds plus 12 months after the last participant’s third visit (Primary Analysis Window). Person-years of follow-up were measured from randomization to the first event of interest (i.e., stage III or IV cancer diagnosis, death, consent withdrawal, or emigration).

Incidence rates were calculated as the number of first eligible events divided by person-years at risk, with 95% confidence intervals (CI) for incidence rates calculated assuming a Poisson distribution. Incidence rate ratios (IRR), 95% CI, and two-sided p-values were calculated using exact Poisson methods. Unstageable cancers and cancers with missing stage (**Supplemental Methods section 7**) were excluded from stage-specific analysis. The primary endpoint was the between-arm comparison of stage III/IV cancer incidence rates after 3 screening rounds and ≥12 months follow-up after the last participant’s third visit. Statistical testing followed a prespecified fixed sequence:

1. 12 prespecified cancer types responsible for >60% of cancer deaths^6,7^: lung, head and neck, colon/rectum, pancreas, myeloma/plasma cell neoplasm, liver/bile duct, stomach, esophagus, anus, lymphoma, ovary, and bladder.
2. All routinely staged cancer types excluding prostate cancer.
3. All routinely staged cancer types.

Testing stopped at the first non-significant step to control type I error. Secondary endpoints (**Supplemental Methods section 6**), including stage IV cancer incidence reduction, were interpreted descriptively with 95% CIs. The width of the CIs are not adjusted for multiple testing and should not be used for hypothesis testing.

### Safety

Adverse events were reported by participants or recorded by qualified trial members. Study-related adverse events were those related to study procedures until NHS referral; “device-related” adverse events occurred after NHS referral with a reasonable temporal possibility of a causal relationship with the onward referral, investigational device, or its use, despite these activities occurring outside of the trial. Adverse events were assessed for relatedness, expectedness, and severity, aligned with the medical safety and monitoring plan (**Supplementary Methods section 8**). Further information on anxiety among those receiving a positive MCED test result,^16^ and radiation exposure and complications associated with diagnostic procedures outside the study, will be published separately.

### Data sharing

Individual-level participant cancer outcome data are not publicly available and cannot be shared by the authors due to confidentiality requirements and restrictions under the applicable data-sharing agreements with National Disease Registry Service (NDRS), an organization within NHS England (formally called the National Cancer Registry and Analysis Service [NCRAS]). Aggregated, anonymized data supporting the findings are provided in the manuscript and supplementary materials.

## Results

### Population

**Figure 1:**
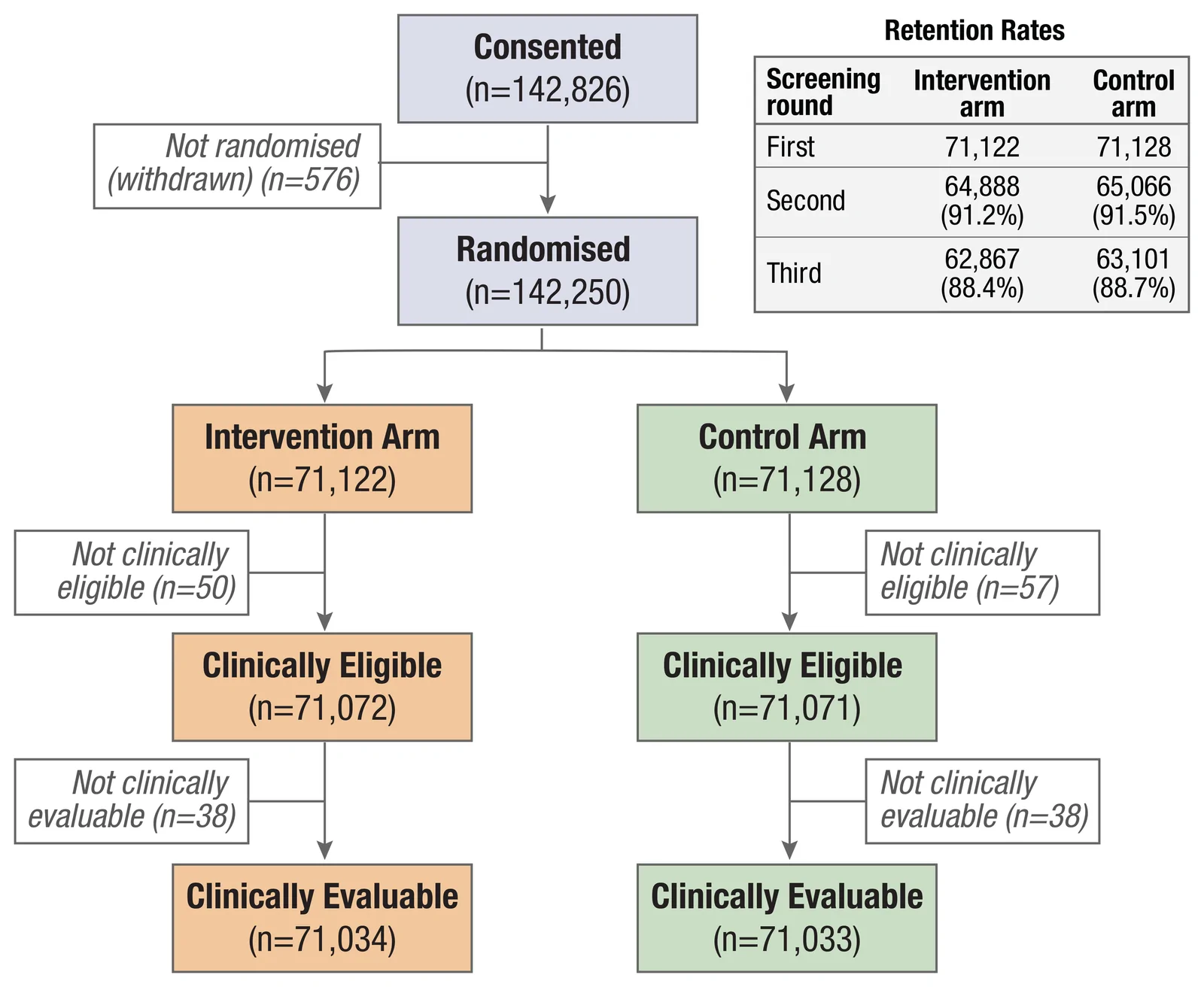
CONSORT diagram for the NHS-Galleri study cohort. Clinically eligible refers to randomized participants who satisfied the inclusion and exclusion criteria. The clinically evaluable analysis set comprised participants who were successfully linked in the routine datasets used in this study.

1,496,311 individuals were invited, and 142,826 participants consented to the trial over 10.5 months^13^; 142,250 (99.6%) had their first blood draw and were randomized to intervention (71,122) or control (71,128) arms (Figure 1). Of the randomized intervention- and control-arm participants, 64,888 (91.2%) and 65,066 (91.5%) returned for their second and 62,867 (88.4%) and 63,101 (88.7%) for their third annual blood draw, respectively. Participants had 12 months follow-up after each of the first two MCED screening rounds, and a median of 17 (range: 12-22) months follow-up after the third round.

By design, participants from the most socioeconomically deprived areas were over-represented (Table 1). Ethnic distribution broadly represented the equivalent-aged English population (Table S3).^13^ Most participants never smoked and reported being up to date on standard-of-care screening. Demographics and characteristics were well balanced between arms at baseline and across screening rounds (Table 1, S4).

### Late-stage cancer outcomes with MCED screening

**Figure 2:**
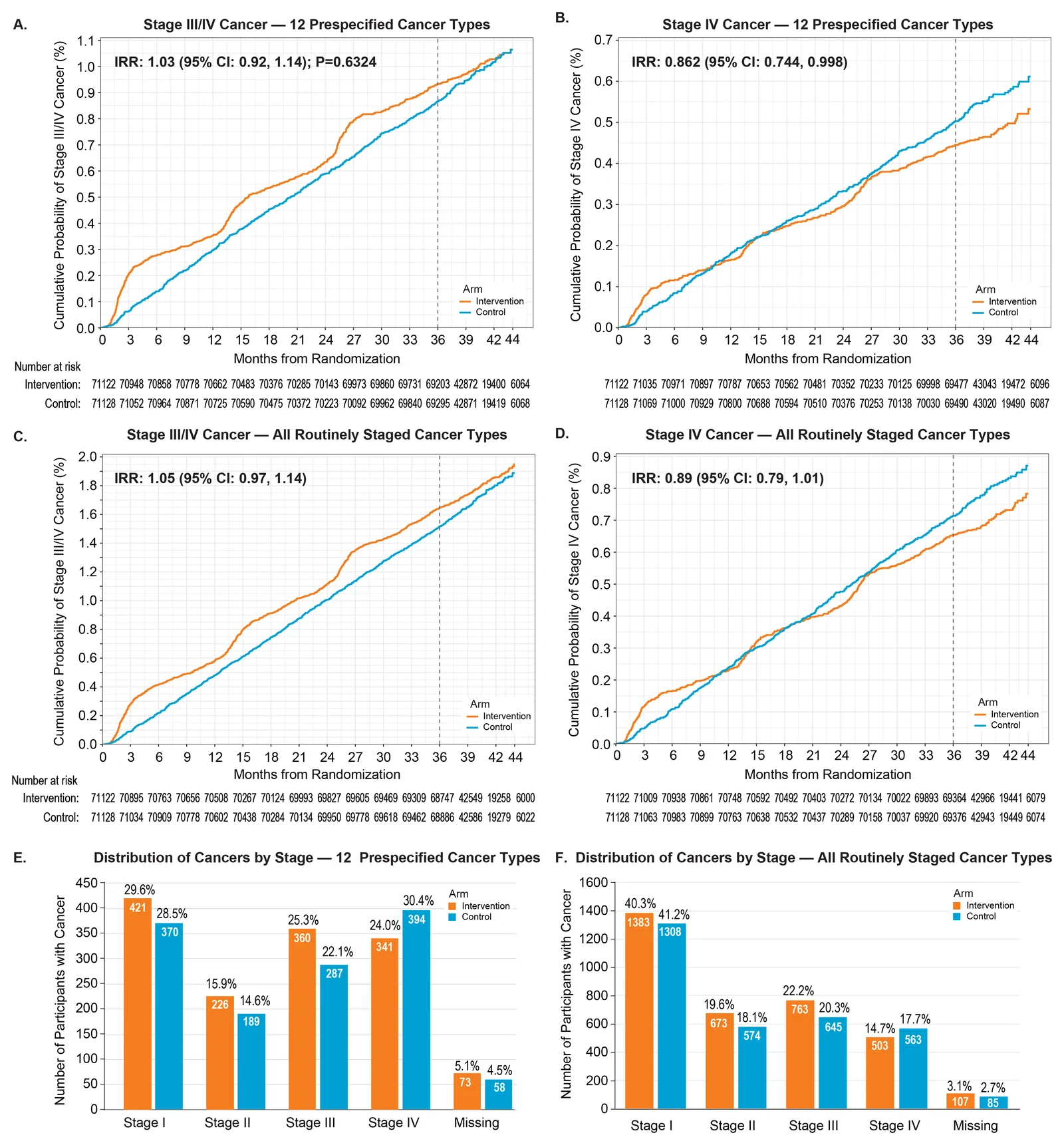
Cumulative probability of stage III/IV cancers and stage IV cancers by trial arm after 3 screening rounds. Cumulative probability of **A**) stage III/IV, 12 prespecified cancer types; **B**) stage IV, 12 prespecified cancer types; **C**) stage III/IV, all routinely staged cancer types; and **D**) stage IV, all routinely staged cancer types (all post-hoc analyses). Distribution of cancers by stage and trial arm shown for **E**) 12 prespecified cancer types (post-hoc analysis) and **F**) all routinely staged cancer types. There were two participants with stage 0 cancer in each of the intervention and control arms. All of these were Paget’s disease, which is invasive at stage 0. IRR, incidence rate ratio.

Overall, 3637 intervention- and 3400 control-arm participants were diagnosed with cancer, of which 3431 and 3177 were routinely staged cancer types; 1421 and 1298 were of the 12 prespecified cancer types (Table S5-S6). There were 706 and 688 participants diagnosed with stage III/IV cancer of the 12 prespecified types in the intervention vs control arm after 3 screening rounds, yielding an incidence rate of 300.5 (95% CI: 278.7-323.5) and 292.5 (271.0-315.1) per 100,000 person-years, respectively (Figure 2, Table 2). Stage III/IV cancer incidence rate did not differ significantly between intervention and control arms (IRR: 1.03 [0.92-1.14], p=0.63). Participant-level descriptive counts (Table S7) revealed an increase in stage III cancers of the 12 prespecified types diagnosed in the intervention (360) vs control (287) arm, outweighing the decrease in stage IV cancers (341 vs 394). The overall result was driven by the higher incidence rate of stage III/IV cancers in the intervention vs control arm in the prevalent round (IRR: 1.19 [0.98-1.43]). In contrast, IRR for stage III/IV cancers in incident rounds comparing intervention vs control were 0.95 (0.77-1.17) for second round (post-hoc); 0.88 (0.73-1.07) for third round (Table S8).

Among the 12 prespecified cancer types, after 3 screening rounds stage IV cancer incidence rate was 145.2 (130.2-161.4) per 100,000 people-years in the intervention arm vs 168.5 (152.3-185.9) per 100,000 people-years in the control arm; IRR: 0.86 (0.74-1.00) (Figure 2, Table 2). In the intervention vs control arm, IRR for stage IV cancers were 0.91 (0.71-1.18) in the prevalent (first) round, 0.78 (0.57-1.06) in the second round, and 0.74 (0.57-0.95) in the third round (Table S9).

The observed IRRs for stage III/IV and IV cancers appeared similar between the 12 prespecified types and all routinely staged types (with and without prostate; Figures 2, S2; Tables 2, S8-S9). Notably, across all routinely staged cancers, IRR for stage IV cancer was 0.78 (0.61-1.00) in the second round and 0.80 (0.65-0.99) in the third round (Table S9).

As a prespecified secondary endpoint, we combined fatal cancers (regardless of stage) with stage III/IV cancers. With this definition, there were 1415 intervention-arm and 1359 control-arm events from all routinely staged cancer types after 3 screening rounds (IRR: 1.04 [0.97-1.12]; Table S10). Excluding cancers with standard-of-care screening (i.e., breast, cervix, colon/rectum), 1077 intervention-arm and 1030 control-arm participants had stage III/IV cancer diagnosed after 3 rounds (IRR: 1.05 [0.96-1.14]; Table S11).

In prespecified exploratory subgroup analyses, the greatest (relative and absolute) differences in stage III/IV and IV cancer rates in the intervention vs control arm were observed among men, those with prior cancer history, and with lower education status; the greatest stage IV decrease among IMD quintiles was observed in the most deprived (Figure S3).

### Increase in early-stage cancers with MCED screening

The observed cancer detection rate in the first round was higher in the intervention arm than the control arm: ratio 1.21 (1.11-1.31; Table S12).

In the third round, the proportion of routinely staged cancers that were stage I/II was 62.8% in the intervention vs 59.9% in the control arm (Table S13). Relatedly, in a post-hoc analysis, after 3 screening rounds, there were 647 stage I/II cancers from the 12 prespecified types in the intervention arm and 559 in the control arm (relative risk: 1.16 [1.03-1.30]) and 1007 vs 846 for stage I/II/III cancers (relative risk 1.19 [1.00-1.30]; Table S14). Similar results were observed for participants with all routinely staged cancer types (with and without prostate; Table S14).

### Route to cancer diagnosis

Based on the ‘route to diagnosis’ recorded by the cancer registry, in the intervention vs control arm, there was an 18.9% (1775 vs 1493) increase in diagnoses following urgent cancer referral (the referral route for positive MCED test results)^14^ and a 21.3% (225 vs 286) decrease in emergency presentations after 3 screening rounds (Table S5).

**Figure 3:**
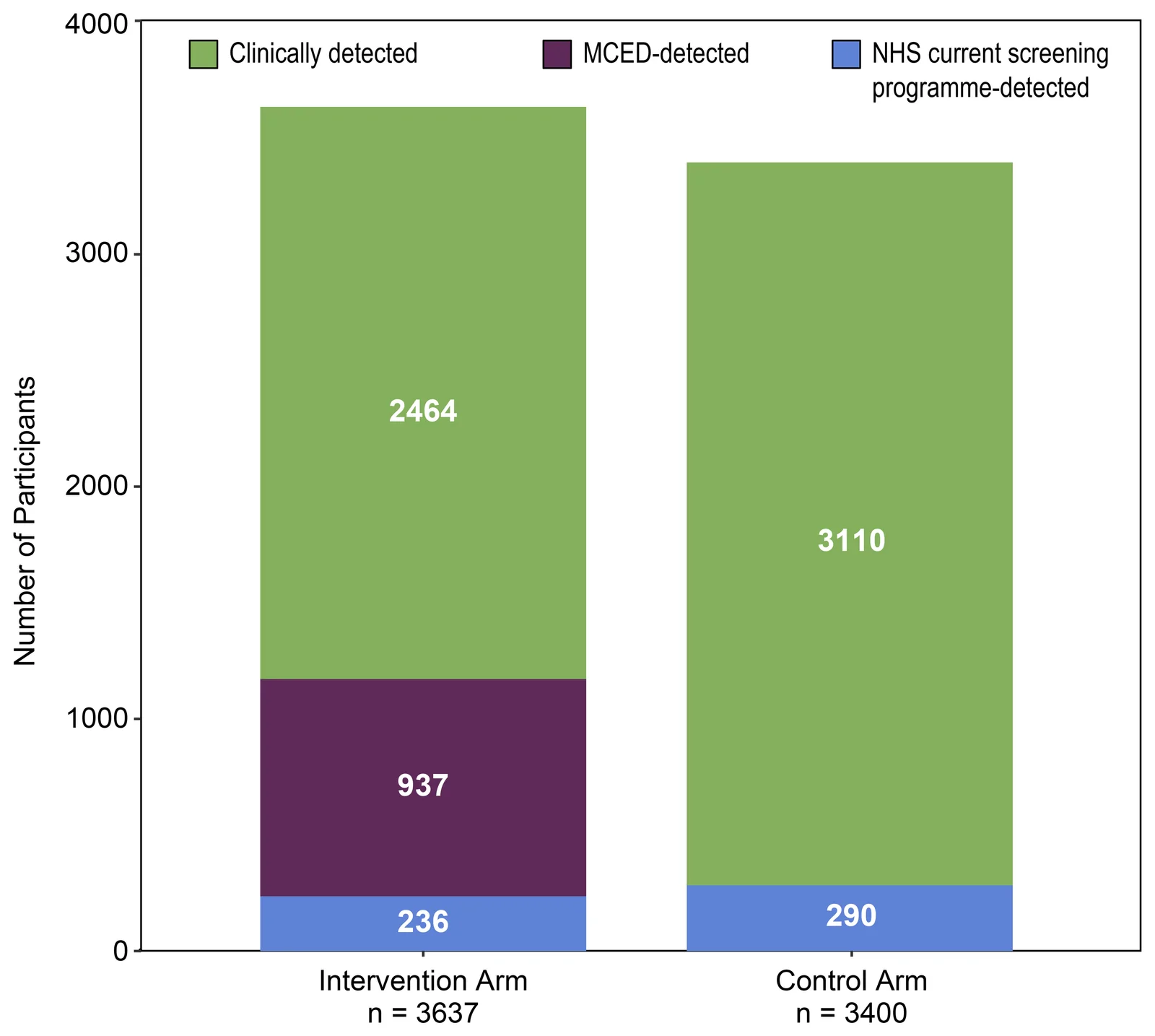
Distribution of the route to cancer diagnosis by trial arm after 3 annual screening rounds. For participants with multiple cancers diagnosed, only the route to diagnosis of the first cancer is included.

In the intervention arm, 1173/3637 (32.3%) participants had screen-detected cancers: 937/3637 (25.8%) following a positive MCED test result and 236/3637 (6.5%) by NHS screening programs (Figure 3). In the control arm, 290/3400 (8.5%) had cancer detected by NHS screening programs. Thus, adding MCED to standard-of-care screening was associated with an apparent increase in screen-detected cancers (1173 vs 290) and in screening program sensitivity (32.3% vs 8.5%; Table S15).

There were 20.8% fewer clinically detected cancers (i.e., cancers neither screen-detected nor MCED-detected): 2464/3637 (67.7%) vs 3110/3400 (91.5%) in the intervention vs control arm (Figure 3). Similar results were observed across all 3 screening rounds (Figure S4).

### Safety

Across 3 screening rounds, 371 (0.52%) randomized intervention-arm and 321 (0.45%) randomized control-arm participants had related adverse events; none were serious (Tables S16-S17). The excess was primarily from anxiety following receipt of a positive MCED result. Among intervention-arm participants, 337 (0.47%) had study-related adverse events, 27 (0.04%) had device-related adverse events, and 11 (0.02%) had study- and device-related adverse events (Table S16). Among control-arm participants, 321 (0.45%) had study-related adverse events. The most common study-related adverse events were vessel puncture site bruises, syncope, dizziness, vessel puncture site hematoma, and anxiety.

Participants reported high adherence to standard-of-care cancer screenings (Table S18).

## Discussion

NHS-Galleri is a randomized controlled trial evaluating the clinical utility of an MCED test for population screening. After three annual MCED screening rounds with 12 months follow-up after the last participant’s third visit, we did not observe a reduction in the primary outcome of stage III/IV cancers among the 12 prespecified types. Although the prespecified hierarchical plan to adjust for multiple testing precluded any formal testing of secondary outcomes, the results suggest the possibility that the intervention was associated with fewer stage IV cancer diagnosed in incident rounds, in which observed trends would be expected to reflect those of a stable screening program with a routinely screened population. Stage IV cancer reduction is clinically important since stage IV disease confers worse survival than earlier-stage.^1^

The observed lower rate of stage IV cancer was offset by an increase in stage III cancers, resulting in no overall reduction in stage III/IV combined. Prevalent-round stage III cancers appeared to be the predominant driver of the missed primary endpoint for the 12 prespecified types, with the higher number in the intervention arm potentially reflecting several non-mutually exclusive mechanisms, including earlier detection of cancers that might otherwise have presented at stage IV, and longer than anticipated lead-time effects. Additional follow-up will provide more evidence about the intervention’s effects on long-term III/IV incidence. It is possible that the full clinical utility of MCED screening will emerge over time, after depletion of the prevalent pool, consistent with published screening studies.^17–20^ Longer follow-up is needed to determine whether the excess of stage III cancers is transient and the observed stage distribution translates into lower cancer-specific mortality.

Late-stage cancer incidence was chosen as the primary endpoint because it is clinically relevant and can be assessed earlier than cancer-specific mortality, although its value as a surrogate may vary by cancer type and screening context.^21–23^ A favorable result requires a lower late-stage cancer incidence rate, not merely more early-stage diagnoses or a lower late-stage proportion, which could reflect overdiagnosis without reducing advanced disease. At trial design, microsimulation enabled sample-size planning despite limited evidence on cancer dwell times and preclinical ctDNA detectability. These simplified assumptions projected a larger and quicker stage shift than observed. Subsequent model development has considered broader sojourn-time ranges and lower screening sensitivity.^24^ Further analyses incorporating trial data should better characterize these parameters and inform the design of future studies. Cancer-specific and nested mortality will be analyzed at 3 and 6 years after final trial visit as secondary endpoints; the trial was not powered for mortality endpoints.

The intervention arm had more cancers diagnosed at earlier stages than the control arm. The current analysis cannot distinguish between more cancers being found before the equivalent cancers are diagnosed in the control arm versus overdiagnosis. Given that cfDNA shedding is a hallmark of more aggressive cancers, these excess cancers may be more indolent. Recent evidence suggests that cancers detected by circulating tumor DNA-based MCED tests are unlikely to be over diagnosed, as survival outcomes in case control studies in participants with positive MCED results were consistent with population averages, even in early stages.^25^ Rather, the excess stage I/II cancers diagnosed in the intervention arm are likely due to earlier detection of clinically significant disease. Longer follow-up will help address these questions. Future analysis to assess overdiagnosis is planned and will be published separately.

The addition of MCED testing increased the number of screen-detected cancers and decreased the number of clinically detected cancers. Diagnoses via emergency presentations were reduced by 21.3%; such diagnoses are associated with poorer survival, even for the same cancer type and stage.^2,26^

The performance characteristics of the MCED test are the subject of another publication.^27^ Briefly summarized, positive MCED tests were obtained in 722 (1.03%) in the first round, 518 (0.80%) in the second round, and 561 (0.90%) in the third round. Of these 1801 positive MCED tests, 937 (52%) were found to have cancer; of these, 815 (87.0%) were located in the first-ranked predicted site of origin predicted by the positive MCED.

The NHS-Galleri trial is a randomized controlled trial designed to evaluate an MCED test at a population level, providing robust evidence on the implementation of this approach in routine screening settings. The findings from this trial are consistent with those from the prospective PATHFINDER 2 study.^9^ The fact that these two independent prospective datasets demonstrate reproducibility across different healthcare settings (UK and US) supports the feasibility of implementing this MCED screening at scale. Future analyses of health economics and outcomes research endpoints that provide further insights will be published separately.

### Strengths

Trial strengths included the randomized, double-blind design, ascertainment of outcome data from a quality-assured national cancer registry, socioeconomically diverse representation of the target population, high retention throughout the trial, and the use of existing standardized diagnostic pathways within the NHS,^14^ ensuring clinical management as close to usual care as possible. These features reduced bias and supported generalizability of the trial results.

### Limitations

The follow-up was relatively short for a screening trial and cancer incidence between the two arms has not yet converged, which suggests the full effects of the MCED intervention may not yet be mature. Continued follow-up is needed to assess how these effects evolve; for example, it is plausible that the excess of stage III cancers in the intervention arm reflects earlier diagnosis of cancers that remain asymptomatic in the control arm and may later present at stage III or IV. The combined stage III/IV primary endpoint may not capture all potentially clinically meaningful effects of screening, such as a shift from stage IV to stage III, diagnosis at an earlier substage within the same stage, and diagnosis before symptom onset. These findings suggest that future screening trials should carefully consider the primary and secondary endpoints including the duration of follow-up after the last screen. Additionally, traditional stage categories should be complemented by outcomes that capture clinically meaningful effects within and beyond stage, such as circulating tumor DNA (ctDNA) fraction. The study was not powered to evaluate a reduction in cancer mortality. The study was not designed to assess different MCED screening intervals or likely participation in a routine MCED screening program. Negative MCED test results were not returned and individual medical records were unavailable, limiting assessment of effects on screening behavior and diagnostic work-up. The study was not powered to assess clinical utility of MCED screening within demographic subgroups or individual cancer types. Adverse events in the trial were systematically collected up until referral into the NHS system for diagnostic workup; thereafter, adverse events were captured only if reported directly by the participant or a clinician. As a result, this trial does not fully characterize the safety profile during diagnostic evaluation following a positive MCED test. This limitation is addressed with the PATHFINDER2 study.^9^

## Conclusion

In this large randomized controlled trial, annual MCED testing added to usual care did not significantly reduce stage III/IV cancers across 12 prespecified types after 3 screening rounds and ≥12 months follow-up. Further follow-up is planned to assess how findings for stage III/IV cancers and the observed difference in stage IV cancers translate to clinical benefit over time.

Disclosure forms provided by the authors are available with the full text of this article at NEJM.org.

## Supplementary Material

Supplementary Appendix

## Figures and Tables

**Table 1:** Demographics and baseline characteristics of the Randomized Analysis set in the NHS-Galleri trial.

		Intervention arm (N = 71,122)	Control arm (N = 71,128)	Total (N = 142,250)
**Median age at randomization, years (IQR)**		66 (59-71)	66 (59-71)	66 (59-71)
**Age groups at randomiz ation, n (%)**	**50-59 years**	18,791 (26.4)	18,889 (26.6)	37,680 (26.5)
	**60-69 years**	28,596 (40.2)	28,792 (40.5)	57,388 (40.3)
	**70-79 years^a^**	23,734 (33.4)	23,447 (33.0)	47,181 (33.2)
**Sex, n (%)**	**Female**	35,723 (50.2)	35,739 (50.2)	71,462 (50.2)
	**Male**	35,399 (49.8)	35,389 (49.8)	70,788 (49.8)
**Race/ethni city, n (%)**	**Asian or Asian British**	2360 (3.3)	2365 (3.3)	4725 (3.3)
	**Black, Black British, Caribbean, or African**	1016 (1.4)	1038 (1.5)	2054 (1.4)
	**White**	66,552 (93.6)	66,566 (93.6)	133,118 (93.6)
	**Mixed^b^**	776 (1.1)	768 (1.1)	1544 (1.1)
	**Other^c^**	318 (0.4)	309 (0.4)	627 (0.4)
	**Missing**	100 (0.1%)	82 (0.1%)	182 (0.1%)
**IMD quintile, n** **(%)**	**1 - Most deprived**	16,071 (22.6)	16,062 (22.6)	32,133 (22.6)
	**2**	13,942 (19.6)	13,980 (19.7)	27,922 (19.6)
	**3**	15,034 (21.1)	14,956 (21.0)	29,990 (21.1)
	**4**	14,372 (20.2)	14,476 (20.4)	28,848 (20.3)
	**5 - Least deprived**	11,469 (16.1)	11,414 (16.0)	22,883 (16.1)
	**Missing**	234 (0.3%)	240 (0.3%)	474 (0.3%)
**Prior history of cancer**	**n (%)**	5328 (7.5)	5355 (7.5)	10,683 (7.5)
	**Median (IQR) time since prior cancer diagnosis-yrs^d^**	10.2 (6.7–15.0)	10.2 (6.7–14.8)	10.2 (6.7–14.9)
**Self-reported screening participati on, n (%)^e^**	**Never heard of cancer screening**	2056 (2.9)	2108 (3.0)	4164 (2.9)
	**Heard of cancer screening but never been invited**	13,955 (19.6)	13,871 (19.5)	27,826 (19.6)
	**Invited for cancer screening but never done it**	861 (1.2)	883 (1.2)	1744 (1.2)
	**Invited for cancer screening, but did not participate every time**	2428 (3.4)	2361 (3.3)	4789 (3.4)
	**Invited for cancer screening and participated every time**	51,013 (71.8)	51,150 (72.0)	102,163 (71.9)
	**Don’t know**	655 (0.9)	608 (0.9)	1263 (0.9)
	**Decline to answer**	51 (0.1)	58 (0.1)	109 (0.1)
	**Missing**	103 (0.1)	89 (0.1)	192 (0.1)
**Smoking status, n** **(%)**	**Currently smokes**	4744 (6.7)	4708 (6.6)	9452 (6.6)
	**Formerly smoked**	27,272 (38.3)	27,148 (38.2)	54,420 (38.3)
	**Never smoked**	39,004 (54.8)	39,187 (55.1)	78,191 (55.0)
	**Missing**	102 (0.1%)	85 (0.1%)	187 (0.1%)
**BMI (kg/m^2^), n** **(%)**	**<25**	23,058 (32.4)	23,203 (32.6)	46,261 (32.5)
	**25–30**	28,009 (39.4)	27,925 (39.3)	55,934 (39.3)
	**≥30.0**	19,739 (27.8)	19,706 (27.7)	39,445 (27.7)
	**Missing**	316 (0.4)	294 (0.4)	610 (0.4)
**History of selected medical diagnoses, n (%)**	**Barrett’s esophagus**	811 (1.1)	810 (1.1)	1621 (1.1)
	**Chronic bowel disease**	1660 (2.3)	1632 (2.3)	3292 (2.3)
	**Chronic liver disease**	1167 (1.6)	1056 (1.5)	2223 (1.6)
	**Diabetes**	7091 (10.0)	7129 (10.0)	14,220 (10.0)
	**Chronic pancreas disease**	304 (0.4)	298 (0.4)	602 (0.4)
	**Systemic lupus erythematosus**	129 (0.2)	140 (0.2)	269 (0.2)
**Education, n (%)**	**School at or before the age of 15 years**	12,672 (17.8)	12,887 (18.1)	25,559 (18.0)
	**CSEs, O-levels, or equivalent**	19,262 (27.1)	19,260 (27.1)	38,522 (27.1)
	**A-levels or equivalent**	6517 (9.2)	6509 (9.2)	13026 (9.2)
	**Further education without a degree**	14,776 (20.8)	14,570 (20.5)	29,346 (20.6)
	**Bachelor’s degree or equivalent**	11,953 (16.8)	11,970 (16.8)	23,923 (16.8)
	**Further degree (e.g., Masters, PhD**	5842 (8.2)	5850 (8.2)	11,692 (8.2)
	**Missing**	100 (0.1)	82 (0.1)	182 (0.1)

BMI, body mass index; IMD, index of multiple deprivation (an area-based measure of relative deprivation); IQR, interquartile range.

a Individuals had to be aged 50-77 years at invitation; however, they could be aged 78 years by enrolment.

b Includes White and Black Caribbean; White and Black African; White and Asian; and any other Mixed or multiple ethnic background.

c Includes Arab and any other ethnic background not listed.

d If a participant had multiple prior cancers, the number of years since the most recent prior cancer to randomisation date is used.

e Percentage is based on the count out of all non-missing responses. Percentages may not sum to 100% across groups due to rounding.

**Table 2:** Stage III/IV and stage IV cancer outcomes after 3 annual screening rounds in the NHS-Galleri trial.

	Prespecified group of 12 cancer types^a^		All routinely staged cancer types excluding prostate cancer		All routinely staged cancer types	
	Intervention arm (N = 71,122)	Control arm (N = 71,128)	Intervention arm (N = 71,122)	Control arm (N = 71,128)	Intervention arm (N = 71,122)	Control arm (N = 71,128)
**Stage III/IV**						
**Participants with stage III/IV cancer, n (%)**	706 (1.0)	688 (1.0)	903 (1.3)	848 (1.2)	1266 (1.8)	1208 (1.7)
**Follow up, person-years**	234,959	235,249	234,667	235,044	234,062	234,495
**Incidence rate per 100,000 person-years (95% CI)**	300.5 (278.7, 323.5)	292.5 (271.0, 315.1)	384.8 (360.1, 410.7)	360.8 (336.9, 385.9)	540.9 (511.5, 571.5)	515.1 (486.5, 545.0)
**Incidence rate difference** **(95% CI)**	8.0 (−23.1, 39.2)		24.0 (−10.9, 59.0)		25.7 (−15.9, 67.4)	
**Incidence rate ratio (95% CI)**	1.03 (0.92, 1.14) p=0.63		1.07 (0.97, 1.17)		1.05 (0.97, 1.14)	
**Stage IV**						
**Participants with stage IV cancer, n (%)**	342 (0.5)	397 (0.6)	412 (0.6)	467 (0.7)	503 (0.7)	563 (0.8)
**Follow up, person-years**	235,584	235,662	235,501	235,585	235,347	235,434
**Incidence rate per 100,000 person-years (95% CI)**	145.2 (130.2, 161.4)	168.5 (152.3, 185.9)	174.9 (158.5, 192.7)	198.2 (180.7, 217.1)	213.7 (195.5, 233.2)	239.1 (219.8, 259.7)
**Incidence rate difference** **(95% CI)**	−23.3 (−46.0, −0.7)		−23.3 (−48.0, 1.4)		−25.4 (−52.6, 1.8)	
**Incidence rate ratio (95% CI)**	0.86 (0.74, 1.00)		0.88 (0.77, 1.01)		0.89 (0.79, 1.01)	

a Lung, head and neck, colon/rectum, pancreas, myeloma/plasma cell neoplasm, liver/bile duct, stomach, esophagus, anus, lymphoma, ovary, and bladder.

Note: In this table for event specific IRR analysis, a participant is counted based on the first qualifying event meeting the corresponding endpoint definition during follow-up. Counts may differ from descriptive analyses tables in which a single cancer/stage is assigned using the highest stage and earliest diagnosis if tied.
