# Supplementary material for: Impact of Multi-Cancer Early Detection Test on Late-Stage Cancer Diagnosis
Source: N Engl J Med. Author manuscript; Available in PMC 2026 Oct 22. (PMC7619610; doi:10.1056/NEJMoa2505723)
Supplement: Supplementary file 1 [file EMS219725-supplement-Supplementary_Appendix.pdf]

Supplementary Appendix

Supplement to: Sasieni P, Johnson P, Round T, et al. Effect of screening with multicancer early-detection test on late-stage cancer diagnosis. N Engl J Med. DOI: 10.1056/NEJMoa2505723

This appendix has been provided by the authors to give readers additional information about the work.

Supplementary Appendix

Table of Contents

Investigators 3
Supplementary Methods 4
1. Complete inclusion and exclusion criteria for the NHS-Galleri Trial. 4
2. Trial oversight and author responsibilities. 4
3. Recruitment methods for the NHS-Galleri Trial. 5
4. Randomization method for the NHS-Galleri Trial. 6
5. Blood draw 8
6. NHS-Galleri trial objectives. 8
7. NHS-Galleri cancer staging definitions, systems, and mapping. 13
8. Adverse event (AE) reporting and capture. 14
9. Analyses not specified in the statistical analysis plan. 15
10. Complete study protocol. 18
11. Complete statistical analysis plan. 18

Supplementary Results 19
Supplementary Figures 19
Figure S1: Study design. 19
Figure S2. Cumulative number of stage III/IV and stage IV cancers by arm after 3 screening rounds. 20
Figure S3. Forest plot of stage III/IV and stage IV reduction by subgroups after 3 screening rounds. 22
Figure S4. Route to diagnosis distribution by arm in (A) prevalent screening round, (B-C) incident screening rounds, and (D) after 3 annual screening rounds. 27
Supplementary Tables 31
Table S1. Number of participants with at least one important protocol deviation by category and trial arm. 31
Table S2. Number of important protocol deviations by category. 32
Table S3. Demographic characteristics of NHS-Galleri trial participants relative to England population. 37
Table S4. NHS-Galleri trial retention in second and third screening round, by arm and demographic characteristics. 40
Table S5: Summary of all cancers diagnosed in the NHS-Galleri trial after 3 screening rounds. 45
Table S6: Distribution of routinely staged cancers by cancer type and stage in the NHS-Galleri trial after 3 screening rounds. 50
Table S7. Distribution of routinely staged cancers by stage across individual screening

rounds. 55
Table S8. Reduction in stage III/IV cancers after each annual screening round in the NHS-Galleri trial. 61
Table S9. Reduction in stage IV cancers after each annual screening round in the NHS-Galleri trial. 67
Table S10. Reduction in advanced cancers after 3 screening rounds. 73
Table S11. Reduction in stage III/IV cancers, excluding breast, cervix and colon/rectum cancers after 3 screening rounds. 76
Table S12. Cancer detection rates in the first (prevalent) screening round. 77
Table S13. Proportion of cancers by stage in the third (incident) screening round. 78
Table S14. Proportion of cancers by stage after 3 screening rounds in the NHS-Galleri trial. 80
Table S15. Program sensitivity for MCED and NHS Screening. 83
Table S16. Safety results by arm: number of participants with related adverse events (AEs) across all 3 screening rounds. 84
Table S17. Safety results by arm: number of related adverse events (AEs) across all 3 screening rounds. 87
Table S18. Self-reported adherence to standard-of-care cancer screening. 90
References **93**

Investigators

**Chief investigators**	Professor Richard Neal, MBChB, University of Exeter<br>Professor Charles Swanton, FRCP PhD, Francis Crick Institute, University College London, and University College London Hospitals<br>Dr Helen Jones, EMS Healthcare
**Co-investigator and clinical trial design/lead statistician**	Peter Sasieni, Professor of Cancer Epidemiology, Co-Lead for the Centre for Cancer Screening, Prevention and Early Diagnosis, Queen Mary University of London

Supplementary Methods

1. Complete inclusion and exclusion criteria for the NHS-Galleri Trial.

Inclusion criteria

1. Participants must be 50-77 years of age, inclusive, at the time of data extraction from NHS datasets or GP records used to identify potential participants; and
2. Participants must be capable of giving signed and legally effective informed consent, which includes compliance with the requirements and restrictions listed in the informed consent form (ICF) and in this protocol. Consent provided by a legally authorized representative was not permitted.

Exclusion criteria

1. Previous or current participation in another GRAIL-sponsored study.
2. Personal history of invasive cancer or hematologic malignancy, diagnosed within the 3 years prior to expected enrollment date.
    a. Note: Individuals with a diagnosis of non-melanoma skin cancer and prostate cancer patients whose only treatment was active surveillance were NOT excluded
3. Definitive treatment for invasive cancer or hematologic malignancy within the 3 years prior to expected enrollment date, including adjuvant hormone therapy for cancer (e.g., for breast or prostate cancer).
4. Currently taking demethylating or cytotoxic agents for any condition.
5. Undergoing current investigation for suspected cancer, defined as having been referred to a two week wait clinic or undergoing investigations at a Rapid Diagnostic Center (RDC) or other clinic with a stated suspicion of cancer.
6. Currently on a palliative care pathway.

2. Trial oversight and author responsibilities.

Trial Registration

The trial was registered with ISRCTN (ISRCTN91431511; submission date: 2 September 2021; registration date: 16 September 2021). The first participant registered occurred on 31 August 2021. A total of 79 participants were registered between 31 August and 1 September 2021 prior to the ISRCTN submission. This was aligned with the ethics approval letter advising the trial be registered within 6 weeks of the recruitment of the first research participant.

Data Access

There were no restrictions placed on the authors' access to the study data included in this manuscript. The authors PS, JW, YL, and WL had access to all de-identified participant-level trial data, including data outside the scope of this manuscript. The authors PS, CS, WL, JW and YL vouch for the completeness and accuracy of the data and for the fidelity of the trial to the study protocol. All authors participated in data interpretation and reviewed, revised, and approved the manuscript.

Author Contributions

Conceptualization: RN, PJ, HK, PS, CS; Data Curation: YL, WL, RS, JW, PS; Formal Analysis: YL, WL, JW, PS; Funding Acquisition: HK; Investigation: HJ; Methodology: RN, PJ, HK, WL, RS, PS, CS; Software: YL, WL, JW; Supervision: RD, SD, HJ, HK, LL, TR, PS, CS; Validation: WL; Visualization: RD, HK, YL, WL, RS, JW, PS, CS; Writing Original Draft Preparation: RD, PJ, HJ, HK, WL, TR, RS, JW, PS, CS; Writing Review and Editing: All authors.

Medical writing support was provided by Emma B. Saxon (Royston, UK), Alexandra L. Thomas, PhD, from Citrus Health Group, Inc. (Chicago, IL), and Ichha Khanal, PhD (West Sussex, UK), and funded by GRAIL, Inc. Graphic design support was provided by Kristi Whitfield from PosterDocs (Oakland, CA) and copy editing support was provided by Erin Spohr from Engage Labs, LLC (Oak Ridge, NJ), both funded by GRAIL, Inc.

A Data Sharing Agreement was in place between NCRAS and GRAIL to govern the release and usage of the NCRAS data. A privacy impact assessment was undertaken with regard to the publication of the data.

3. Recruitment methods for the NHS-Galleri Trial.

Recruitment of individuals aged 50-77 years took place between 31 August 2021 and 16 July 2022. The NHS-Galleri trial aimed to recruit participants across sexes and major ethnic groups, with slightly more from more deprived socioeconomic groups compared with the England population, for a higher event rate.[1] Recruitment of participants aged 50-77 years was from eight of 21 Cancer Alliance regions (area-based NHS organizational units that coordinate cancer care and aim to improve outcomes for patients locally) based on their higher cancer mortality, worse early-stage diagnosis, and greater ethnic diversity.[1]

NHS Digital identified and invited approximately 1.5 million individuals and 142,924 were enrolled in the trial in 10.5 months. Participants were recruited via 3 routes. The first was targeted invitation via the NHS DigiTrials population database, through which the majority of participants were recruited.[1] This database includes all individuals registered with a General Practitioner (GP) in England except those who had opted out, either via the national opt-out

service or from the trial specifically via the NHS DigiTrials website. Briefly, invitation lists of eligible individuals from central patient registration data were generated using a dynamic algorithm that targeted individuals in older and more deprived groups, and were generated weekly following reviews of enrollments. The trial received Health Research Authority approval with support from the Confidentiality Advisory Group (Ref:21/CAG/0056), under Regulation 5 of the Health Service Regulations 2002 ('Section 251 support'), for NHS Digital to send out invitation letters to eligible invitees. The second was direct invitation from GP surgery-based Participant Identification Centers (PICs), which are NHS organizations that can use GP practice records to identify and invite potential trial participants (i.e., those who meet eligibility criteria who have not opted out). The third was 'open enrollment': essentially self-referral, often following engagement activities in a community setting conducted to support enrollment to the trial.[1] Along with these local community-based engagement activities, a national communications campaign was conducted to coincide with the start of the enrollment period.[1]

Seven large and 4 small mobile clinics used to conduct all trial appointments were generally located in socioeconomically deprived areas with good transport links; the larger clinics were stationed in locations with high public footfall, and the smaller mobile clinics were stationed in harder-to-reach areas, e.g., where parking was limited. Efforts were made to reduce potential barriers to participation, e.g., providing language translators, translated materials, and various disabled access measures.[1]

Potential participants were given a participant information sheet and informed consent form, and were offered an initial appointment, at which their eligibility was checked by a qualified trial team member; they then watched a video explaining the trial and provided their consent to participate in the trial.

Participants were reminded of the importance of continuing with standard-of-care NHS screening for which they were eligible, and of informing their GP if any new signs or symptoms arose. All participants received written invitations for subsequent trial appointments and those who had been treated for cancer in the intervening year were advised to speak to a trial nurse to determine whether they should attend.

4. Randomization method for the NHS-Galleri Trial.

After informed consent and baseline peripheral blood sample collection, participants were randomized 1:1 to either having their blood tested with GRAIL's MCED test and positive results returned to the participant (intervention arm), or to having their sample stored for potential future analysis (control arm).

The randomization specification for NHS-Galleri utilizes a permuted random block design (with random block size of 4, 6, or 8), stratified by the 7 mobile clinic units that are deployed across Cancer Alliances in England for participant enrollment and blood collection.

The random seed was defined in the code for reproducibility purposes. Randomization schedules were generated as follows for each stratum:

1. All possible permutations for each block size will be specified. For example, for a block size of 4, there are 6 possible permutations: TTPP, PPTT, TPTP, TPPT, PTPT, PTTP (suppose T represents the intervention and P represents the control). Possible permutations for block sizes of 6 (e.g., one possibility is TPTPTP) and 8 (e.g., TPPTPTTP) will also be specified.
2. A block size will be randomly selected with a probability of 1/3.
3. After a block size is selected, one of the possible permutations of that block size will be selected at random (let us say block Y).
4. Block Y will be added to the schedule to provide randomization assignments for 4, 6 or 8 participants depending on the block size of Y.
5. Repeat steps 1-4 until the desired total number of randomization assignments is obtained.

Randomization assignments contained the variables described below.

Variable name	Possible values
Study Name	NHS-Galleri
Strata Names	NHS_01<br>NHS_02<br>NHS_03<br>NHS_04<br>NHS_05<br>NHS_06<br>NHS_07
Block size	4, 6, 8
Arm assignment	Intervention, Control

To provide sufficient number of assignments in each stratum, sequence length N = 2 * (projected number in each stratum).The needed sequence length for each stratum is listed below.

Stratum_number	N
1	46118
2	46118
3	40628

4	37334
5	37334
6	36236
7	36236

The Biostatistics team wrote the codes and generated the testing sequence to be provided to the Electronic Data Capture (EDC). Two biostatisticians did the coding and independent coding to ensure the accuracy of the codes. A reviewer outside of the Biostatistics team reviewed the codes. A GRAIL team member who was unblinded to the randomization assignment during the course of the study generated the production sequence with a random seed blinded to biostatisticians. The production sequence was blinded to biostatisticians and all other study team staff (except the designated unblinded personnel as required by specific roles responsible for sample management and testing, and systems development).

5. Blood draw

At each blood collection, peripheral blood was obtained by routine venipuncture. Blood was collected in 2 Streck Cell-free DNA BCT tubes (approximately 10 ml of blood in each tube). Blood samples were delivered to a UK pre-analytical laboratory, and intervention arm samples were then sent to GRAIL's CLIA laboratory in the US for plasma isolation where required and cfDNA extraction. The cfDNA samples were processed through the MCED Assay by Clinical laboratory operators according to GRAIL Clinical Laboratory Standard Operating Procedures (SOPs). Any remaining sample and all control samples were stored as plasma without personally identifiable information for consented future research activities relevant to the test.

6. NHS-Galleri trial objectives.

Primary

1. For cancers that are routinely staged, demonstrate a statistically significant reduction in the incidence rate of stage III and IV cancers diagnosed in the intervention arm as compared with the control arm following 3 annual tests and an average of 16–18[a] months follow-up after the third test, using a fixed-sequence strategy as below:
    a. First, evaluate for a statistically significant difference in a prespecified group of primary cancer types: lung, head and neck, colon/rectum, pancreas, myeloma/plasma cell neoplasm, liver/bile duct, stomach, esophagus, anus, lymphoma, ovary, and bladder.

b. If a statistically significant reduction in incidence rate is found, continue by evaluating for a difference in all cancer types excluding prostate cancer.

c. If the above evaluations are both significant, evaluate for a difference in all cancer types.

Secondary

1. For cancers that are routinely staged, demonstrate a statistically significant[b] reduction in the incidence rate of advanced cancers (defined as a cancer diagnosed at stage III or IV or one that results in a cancer-specific death)[c] in the intervention arm as compared to the control arm following 3 annual tests and an average of 16-18[a] months follow-up after the third test, using a fixed-sequence strategy as below:

    a. First, evaluate for a statistically significant difference in a prespecified group of primary cancer types: lung, head and neck, colon/rectum pancreas, myeloma/plasma cell neoplasm, liver/bile duct, stomach, esophagus, anus, lymphoma, ovary, and bladder.

    b. If a statistically significant reduction in incidence rate is found, continue by evaluating for a difference in all cancer types excluding prostate cancer.

    c. If the above evaluations are both significant, evaluate for a difference in all cancer types.

2. For cancers that are routinely staged, demonstrate a difference in the proportion of stage I and II cancers between the 2 arms of the study in the third testing round (i.e., for cancers diagnosed within 12 months of follow-up after the third test).

3. Evaluate the performance (overall sensitivity, specificity, positive predictive value [PPV], negative predictive value [NPV] and cancer signal origin accuracy) of the MCED test in the intervention arm after each annual round of testing and in aggregate across the 3 intervals of testing.

4. Evaluate the safety, including harms, of the testing pathway in the intervention arm among individuals with positive MCED test results.

    a. Assess participant-reported psychological impact, including anxiety, among those receiving a positive MCED test result following the initial blood draw at various time points.

    b. Examine the radiation exposure by participants associated with follow-up diagnostic procedures following a positive MCED test result.

    c. Describe the number and type of invasive procedures performed, and number of complications and deaths associated with follow-up diagnostic procedures.

5. Demonstrate a statistically significant reduction in the incidence rate of stage IV cancers diagnosed in the intervention arm compared to the control arm, following the initial screening round and 12 months of follow-up (excluding cancers identified by the test performed at the 12-month visit):

    a. For a prespecified group of cancer types: lung, head and neck, colon/rectum, pancreas, myeloma/plasma cell neoplasm, liver/bile duct, stomach, esophagus, anus, lymphoma, ovary, and bladder;

    b. For all cancer types excluding prostate cancer;

    c. For all cancer types.

6. Demonstrate a reduction in the incidence rate of stage IV cancers diagnosed in the intervention arm compared to the control arm, following 3 annual tests and an average of 16-18[a] months follow-up after the third test:

    a. For a prespecified group of cancer types: lung, head and neck, colon/rectum, pancreas, myeloma/plasma cell neoplasm, liver/bile duct, stomach, esophagus, anus lymphoma, ovary, and bladder;

    b. For all cancer types excluding prostate cancer;

    c. For all cancer types.

7. Demonstrate a statistically significant difference in cancer detection rates in the 2 arms of the study with 12 months follow-up after the first test.

8. Model the expected differences in cancer-specific mortality at 7 years post-randomization based on cancers diagnosed within an average of 40-42[a] months of randomization.

9. Summarize stage distribution per cancer type for the 2 arms, and compare the incidence rate of stage IV alone as well as stage III and IV cancers, following the third screening and an average 16-18[a] months of follow-up.

10. Demonstrate a reduction in the incidence rate of stage III and IV cancers, excluding breast, cervix, and colon/rectum (i.e., those without a standard-of-care screening

modality) as compared to the control arm following the third annual screening and an average 16-18[a] months of follow-up.

11. Assess the impact on cancer-specific mortality:
    a. (Endpoint powered) assess the potential for avoidance/postponement of cancer death by comparing the rates of death from cancer with a positive MCED test at any of the 3 screening rounds in the intervention arm with that among individuals retrospectively testing positive at any of the 3 screening rounds in the control arm, at 3 years after the last study visit (retrospectively testing all samples from cancer deaths in the control arm).
    b. (Endpoint not powered) compare cancer-specific mortality in the intervention and control arms for all cancer types at (i) 3 years and (ii) 6 years after the last study visit.
12. Compare the incidence rate of stage IV cancers following the second blood draw and 12 months of follow-up, with prevalent cases excluded.
13. Compare the incidence rate of stage III and IV cancers following the third blood draw and an average 16-18[a] months of follow-up, with cases from the first and second screenings excluded.
14. Assess the impact of use of the MCED test across 3 annual timepoints on healthcare resource utilization for cancer diagnosis and treatment.
15. Assess the potential impact of overdiagnosis by comparing the cumulative incidence (Kaplan-Meier curves) of cancers diagnosed after a positive baseline MCED test up to 36-48 months after randomization between the intervention and control arms (retrospectively testing baseline samples from all participants diagnosed with cancer in the control arm).

Exploratory[d]

1. Assess any reduction of cancer of unknown primary (CUP) observed in the intervention arm compared with the control arm.
2. Retrospectively test the participants in the control arm who were diagnosed with a cancer of unknown primary (CUP) and report the cancer signal of origin detected by the MCED test.

3. Assess the primary and secondary objectives in clinically meaningful subsets (e.g., by age, gender, socioeconomic status, risk factors at enrollment, prior cancer history at enrollment, ethnicity).
4. In the intervention arm, retrospectively assess the interval cancer rates at 0 to <4 months, 4 to <8 months, and 8 to <12 months following a negative test and at 12 to <16 and 16 to <20 months after the third annual screen.
5. Compare cancer-specific mortality in the intervention and control arms following the third screening and an average 16-18[a] months of follow-up, for a prespecified group of cancer types: lung, head and neck, colon/rectum, pancreas, myeloma/plasma cell neoplasm, liver/bile duct, stomach, esophagus, anus, lymphoma, ovary, and bladder.
6. Assess any potential impact of a positive MCED test on non-cancer mortality and all-cause mortality with a positive MCED test at any of the 3 screening rounds at 3 years after the last study visit:
    a. Compare MCED test-positive non-cancer deaths between the 2 arms (retrospectively testing all samples from non-cancer deaths in the control arm).
    b. Compare MCED test-positive all-cause mortality between the 2 arms (retrospectively testing all samples from all deaths in the control arm).
7. Describe the lymphoma subtypes diagnosed as a result of the MCED testing pathway and compare detection rates across subtypes.

[a]Enrollment took place over 10.5 months. Follow-up for cancer diagnoses after the third test continued until 12 months after the last participant's third visit for an average of 16-18 months of follow-up after the third test or 40-42 months from randomization. Follow up of cancer diagnoses continued for all participants even if some missed one or both of their second or third blood draws.

[b]Although the NHS-Galleri Study Protocol and Statistical Analysis Plan both describe statistical significance testing for certain secondary endpoints, no formal hypothesis testing beyond the primary endpoint was planned or conducted to avoid type I error inflation due to multiplicity. All secondary endpoint analyses are descriptive in nature.

[c]Secondary objective 1 is a sensitivity analysis of the primary objective, with a broader definition of 'advanced cancer'.

[d]Unless stated otherwise, all exploratory objectives are to be assessed at an average of 16-18 months of follow-up after the third blood draw.

7. NHS-Galleri cancer staging definitions, systems, and mapping.

Routinely staged cancer

Registry stage (stage at diagnosis) was defined as the stage assigned by the National Disease Registration Service (NDRS) from all information available to the cancer registry, including clinical, pathological, and other routinely collected cancer registration data. It may correspond to clinical or pathological stages. For the primary analysis, 'routinely staged cancer' included cancers that were staged based on the following staging systems: Tumor Node Metastasis (TNM), International Federation of Gynecology and Obstetrics (FIGO), Ann Arbor, European Neuroendocrine Tumor Society (ENETS) and International Staging System (ISS). Brain cancers, leukemias, and cancers of unknown primary are not routinely staged, thus are not included.

Staging for routinely staged cancer

For analysis purposes, the staging for routinely staged cancer was classified as below.

**NHS-Galleri Stage**	Stage I	Stage II	Stage III	Stage IV
**Definition**	TNM I<br>FIGO I<br>Ann Arbor I<br>ISS I<br>ENETS I	TNM II<br>FIGO II<br>Ann Arbor II<br>ISS II<br>ENETS II	TNM III<br>FIGO III<br>Ann Arbor III<br>-[a]<br>ENETS III	TNM IV<br>FIGO IV<br>Ann Arbor IV<br>ISS III[a]<br>ENETS IV

[a]For ISS, stage III represents the most advanced stage in the staging system, thus it is mapped to the most advanced stage for the routinely staged cancers.

Stageable cancer

Stageable cancers included those for which an internationally recognized staging system existed for the morphology and site (topography) combination. These combinations were based on the suggested sites and morphologies listed in the Union for International Cancer Control (UICC) TNM classification and published in the UK and Ireland Association of Cancer Registries (UKIACR) performance indicators.

Unstageable cancer

Unstageable cancers included those for which a staging system did not exist for the morphology and site (topography) combination.

Missing cancer stage

Cancers with missing stage were those with a valid morphology and site combination within a staging system, but for which stage information was not present or was partial. It was possible that the staging procedure was not undertaken, or the data were not captured adequately; stage was therefore missing due to a lack of submitted data.

8. Adverse event (AE) reporting and capture.

Adverse events (AEs), were collected, documented, analyzed, and reported. The definition of an AE was ‘any untoward medical occurrence in a patient or study participant, temporally associated with a study procedure, whether or not considered related to the study procedures’. An AE could therefore be any unfavorable and unintended sign (including an abnormal laboratory finding), symptom, or disease (new or exacerbated) temporally associated with a study procedure.

A serious adverse event (SAE) was defined as ‘an untoward occurrence that: (1) resulted in death; (2) was life-threatening; (3) required hospitalization or prolongation of existing hospitalization; (4) resulted in persistent or significant disability or incapacity; (5) consisted of a congenital abnormality or birth defect; or (6) was otherwise considered medically significant by the Chief Investigator’.

Potential AEs could be reported by the participant (or, where appropriate, by a caregiver or surrogate), and/or observed and reported by a study team member. There was an unblinded team, including data management and medical monitor who were responsible for participant safety including assessing AEs. AEs were recorded in the EDC at each study visit. The main risks in this trial were those associated with venipuncture; potential AEs included temporary discomfort and a minimal risk of slight bleeding, bruising, lightheadedness, and, in rare instances, fainting. Other AEs consistent with the published literature on the complications of venipuncture were considered expected. Qualified staff at the blood collection centers monitored each participant for the occurrence of AEs at each study visit.

SAEs were reported to the sponsor or designee, usually within 24 hours of the study site being aware. Once AEs were entered into the EDC, they were assessed for seriousness and reportability by a medically qualified investigator or appropriately qualified designee and the sponsor medical monitor.

Any AE that was assessed as mild/moderate was not required to be followed up. Any AE assessed as severe/serious and related to the study/device was followed up for resolution, and up to 3 attempts to contact the participant were usually made. All serious/severe AEs, whether considered related or unrelated, that remained ongoing were reviewed with the Chief Investigators and the medical monitoring team to decide whether further information was needed, or whether the AE information could be finalized.

AEs reported to the study team that occurred outside of study procedures/visits were recorded, evaluated, and reported to the appropriate authorities in accordance with applicable requirements, detailed in relevant standard operating procedures. This included those for post-market surveillance and vigilance reporting, as the Galleri test is a CE-marked and/or UKCA-marked product. AEs occurring after the point of referral (after the end of the study procedures) could be reported by participants or clinicians to the NHS-Galleri trial customer service team; they were assessed for their relationship to the device and were classified as device-related AEs where appropriate. The term 'device-related' was used in this study to indicate an AE that was reported outside of protocol-defined study procedures in order to identify AEs that may have occurred in the setting of MCED testing and any subsequent evaluations.

Cancers were not captured as AEs, as they were study endpoints, in line with FDA guidance (Investigator Responsibilities - Safety Reporting for Investigational Drugs and Devices).[2]

9. Analyses not specified in the statistical analysis plan.

The following post-hoc analyses were undertaken.

Program sensitivity for MCED screening

Program sensitivity for screening was calculated as the proportion of cancers detected through screening pathways, corresponding to all cancers diagnosed in participants with a positive MCED test result, all cancers diagnosed via NHS Standard of Care Screening pathways, divided by the total number of cancers diagnosed.

Cumulative probability of stage III and IV and stage IV cancer

Post-hoc analyses were conducted to generate Kaplan-Meier (KM) curves estimating the cumulative probability of progression to stage III and IV cancer and stage IV cancer. These KM curves provide a more straightforward interpretation of the data compared to figures of cumulative numbers. As all participants were administratively censored on the same calendar date, there was variable follow-up depending on the participant's randomization date over the 10.5-month period.

Early-stage cancers in the primary analysis window for the prespecified group of 12 cancer types

The absolute difference in proportions of stage I/II cancers between the intervention and control arms was a prespecified secondary endpoint. Post-hoc descriptive analyses were performed to calculate the proportion of early-stage cancers in the primary analysis window for the prespecified group of 12 cancer types. Relative risks and 95% confidence intervals (to support interpretation of the same stage I/II endpoint) were calculated using the Wald method on the log scale under binomial sampling assumptions.

Reduction of stage III/IV cancer incidence in first (prevalent) screening round

Post-hoc analysis was conducted to compare the incidence of stage III/IV cancer between the intervention and control arms in the first screening round. Incidence rates and corresponding 95% confidence intervals for each arm were calculated using exact Poisson (Garwood) methods. The 95% confidence interval for the incidence rate difference (IRD) was derived using the Method of Variance Estimates Recovery with Jeffreys equal-tailed single-rate intervals. The Jeffreys-based MOVER approach was selected for the between-arm comparison because the direct combination of 2 Garwood intervals does not guarantee nominal coverage for a difference, whereas MOVER-J maintains near-exact coverage and balanced precision.

Reduction of stage III/IV cancer incidence in second (incident) screening round

Similar methods as above were used for a post-hoc analysis to compare the incidence of stage III/ IV cancer between the intervention and control arms in the second screening round.

NCRAS review of participants with a positive MCED test result diagnosed via emergency presentation

All participants with a positive MCED test result were referred per protocol by trial nurses to urgent cancer referral pathways (also known as Two Week Wait) for investigation and diagnosis. Details of how NHS England's established referral pathways were leveraged in the trial have been described previously.[3]

The GRAIL study team identified potential data discrepancies in the Cancer Registration data for several participants who had a positive MCED test result, but did not have an urgent cancer referral as their route to diagnosis. The GRAIL study team requested an independent NCRAS analyst to conduct a targeted review of 39 participants with a positive MCED test result who were diagnosed via emergency presentation; this was not a systematic review of route to diagnosis data for all cancer cases. This required the analyst to be unblinded only to the limited information needed for the review.

Given the targeted nature of the review by NCRAS, this analysis should be considered a supportive sensitivity analysis rather than the intention-to-treat randomized arm comparison, as described in the secondary objective. It is possible that some emergency presentations in the control arm have also been misclassified.

Typically, patients are referred via their GP into an urgent cancer referral pathway. It is possible the potential data quality issue arose because trial nurses referred NHS-Galleri participants directly into NHS services rather than via their GP. The route to diagnosis associated with a cancer registration is algorithmically assigned and the information required to assign urgent cancer referral as the route to diagnosis may not have been captured during the referral. Although the targeted review only included participants with a positive MCED test result who had

emergency presentation recorded as their route to diagnosis, the same data quality issue may also affect participants with a positive MCED test result who were recorded with other non-urgent cancer referral routes to diagnosis.

10. Complete study protocol.

Please see the file uploaded on www.nejm.org.

Protocol deviations in the trial were recorded and are summarized in Tables S1 and S2.

11. Complete statistical analysis plan.

Please see the file uploaded on www.nejm.org.

Supplementary Results

Supplementary Figures

Figure S1: Study design.

An overview of the NHS-Galleri trial.[4]

MCED, multi-cancer early detection; NHS, National Health Service.

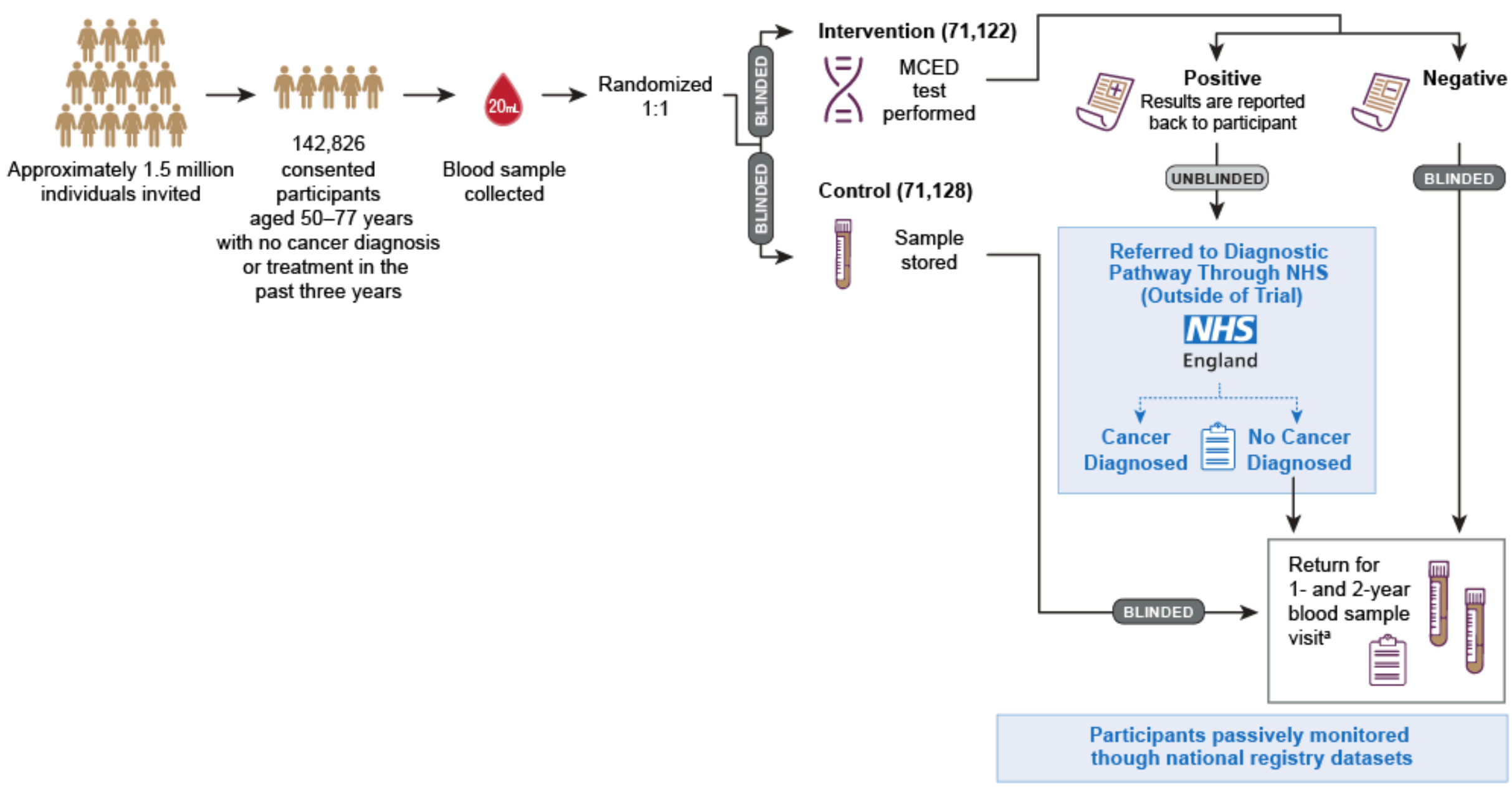

[a]Participants who were diagnosed with cancer were not required to return for blood sample collection.

Figure S2. Cumulative number of stage III/IV and stage IV cancers by arm after 3 screening rounds.

Cumulative probability of A) stage III/IV and B) stage IV for all routinely staged cancer types excluding prostate cancer (post-hoc analyses). C) Distribution of cancers by stage and arm were shown for all routinely staged cancer types except prostate cancers (post-hoc analysis). There were 2 participants with stage 0 cancer in each of the intervention and control arms. All of these were Paget's disease, which is invasive at stage 0.

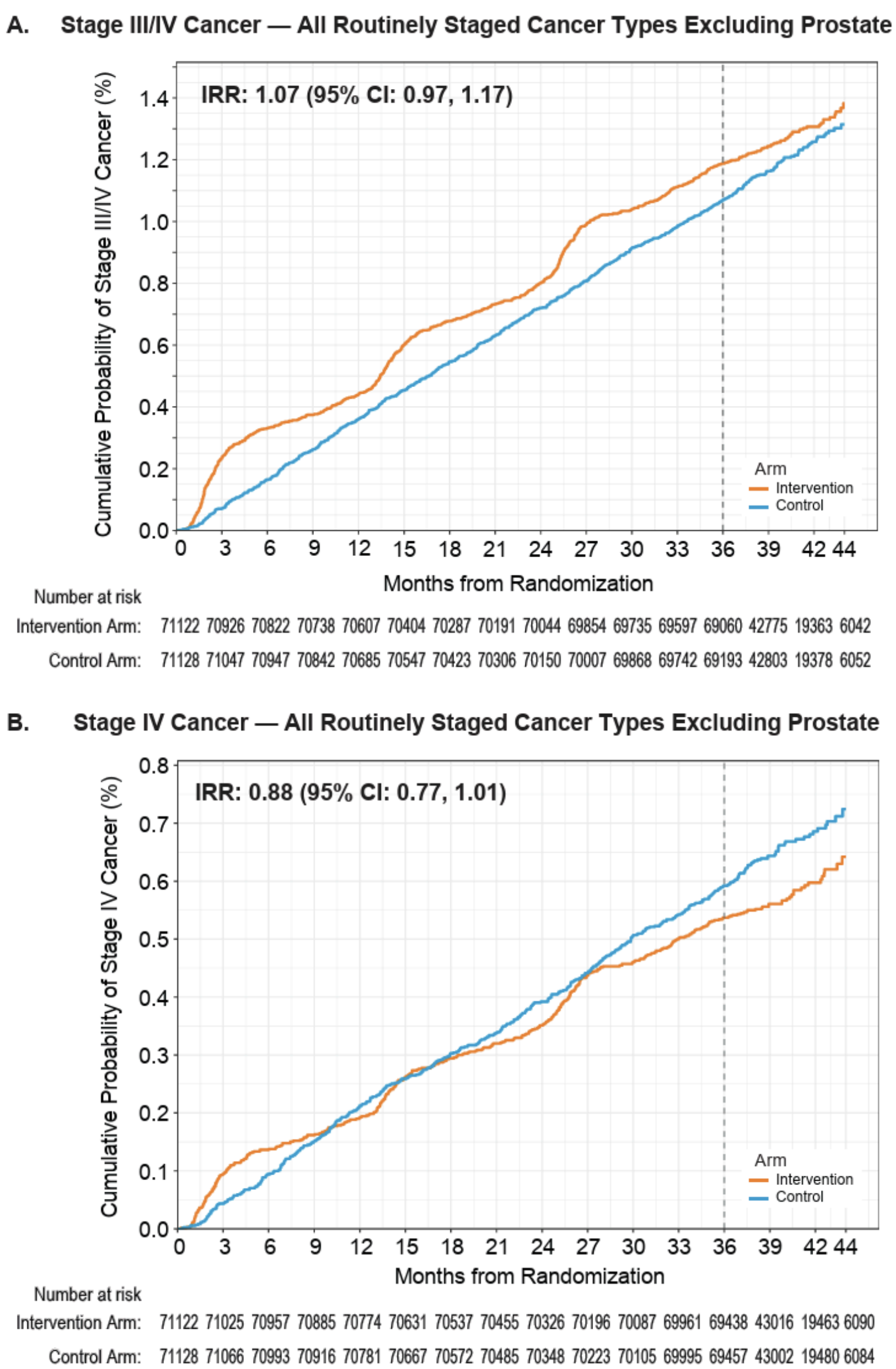

C.

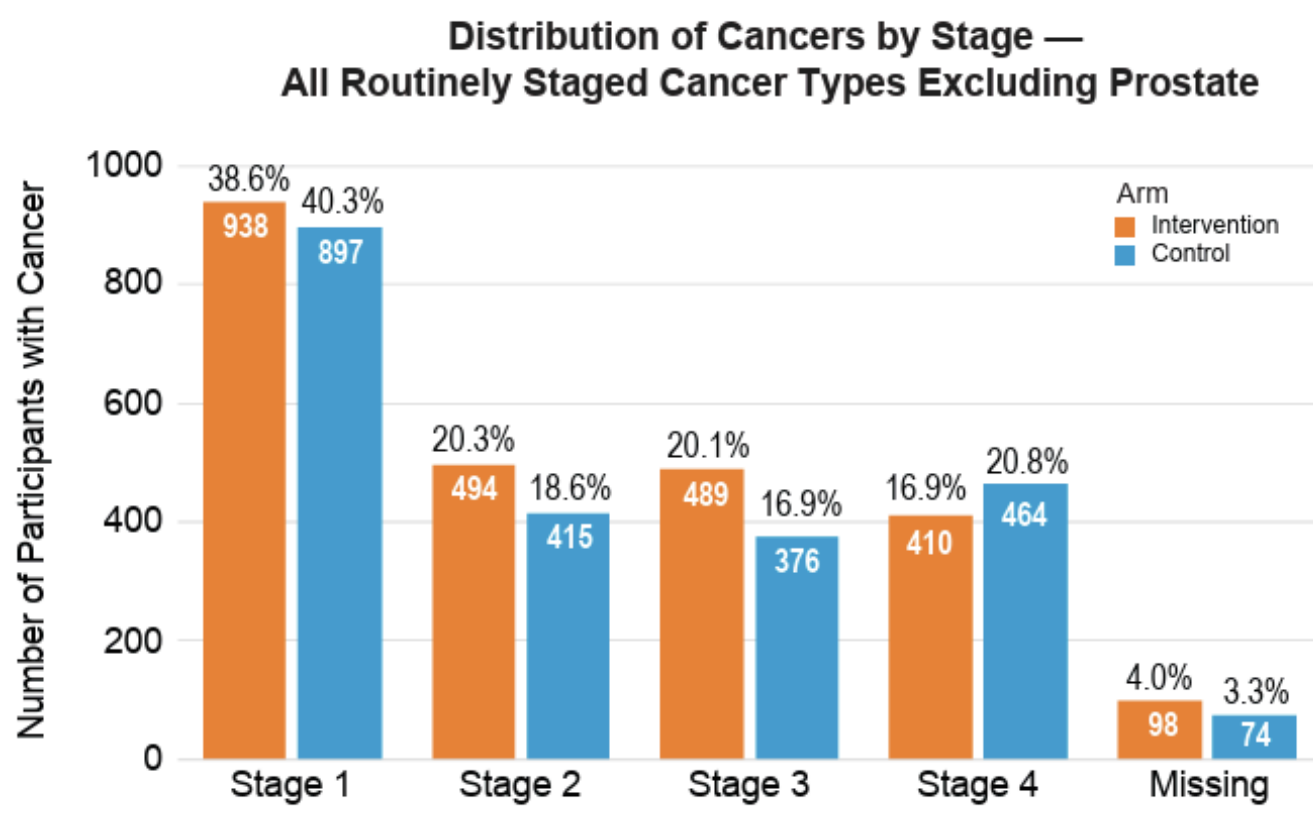
Distribution of Cancers by Stage —
All Routinely Staged Cancer Types Excluding Prostate
Number of Participants with Cancer
1000
800
600
400
200
0
38.6%
938
40.3%
897
20.3%
494
18.6%
415
20.1%
489
16.9%
376
16.9%
410
20.8%
464
4.0%
98
3.3%
74
Stage 1
Stage 2
Stage 3
Stage 4
Missing
Arm
Intervention
Control

Figure S3. Forest plot of stage III/IV and stage IV reduction by subgroups after 3 screening rounds.

Subgroup analysis for age group (50-59, 60-69, 70+) A) stage III/IV, B) stage IV; quintiles of IMD C) stage III/IV, D) stage IV; sex E) stage III/IV, F) stage IV; race and ethnicity G) stage III/IV, H) stage IV; smoking status I) stage III/IV, J) stage IV; prior cancer history K) stage III/IV, L) stage IV; education M) stage III/IV, N) stage IV. Subgroup analyses should be interpreted cautiously as results are variable and underpowered.

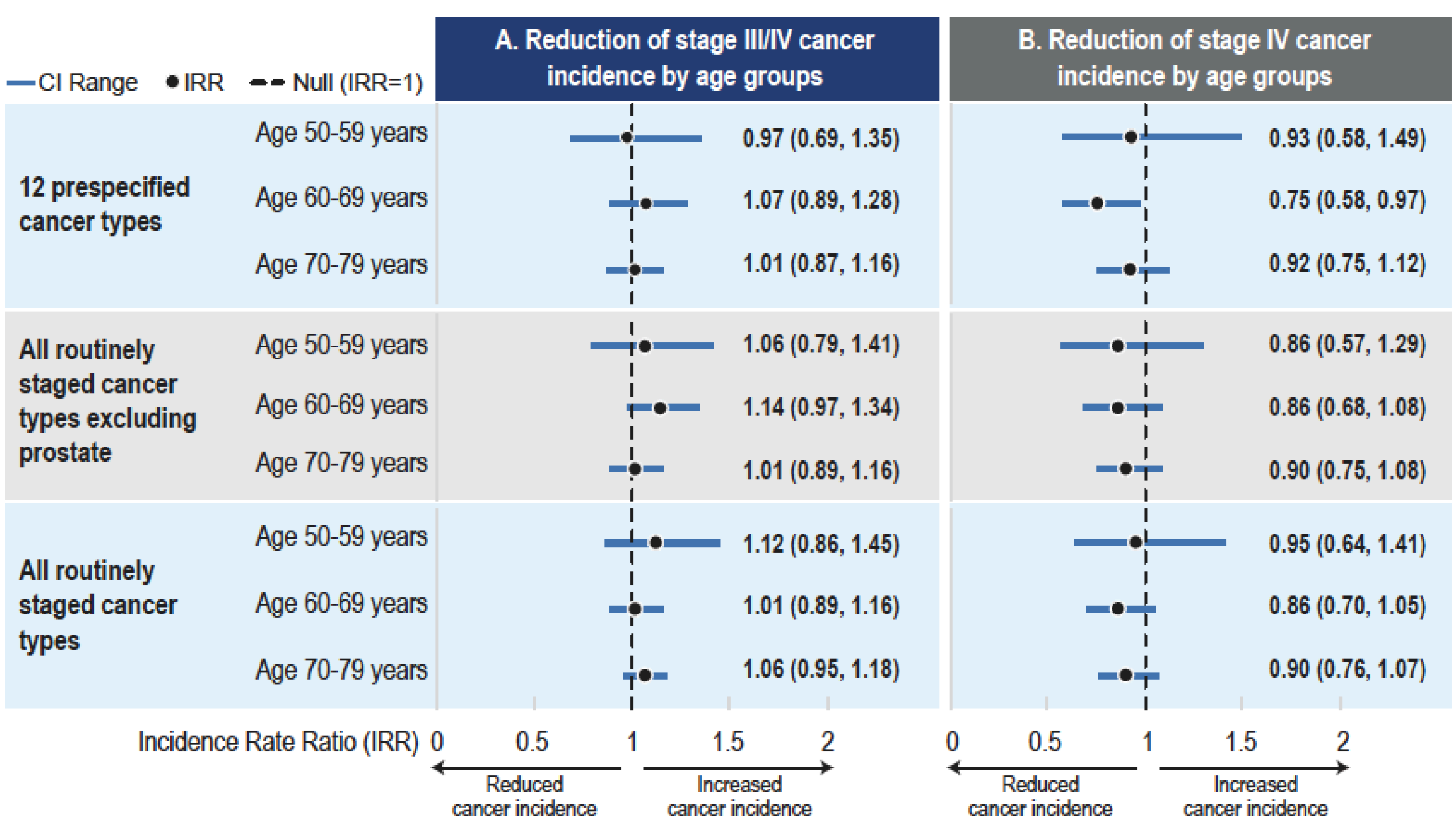

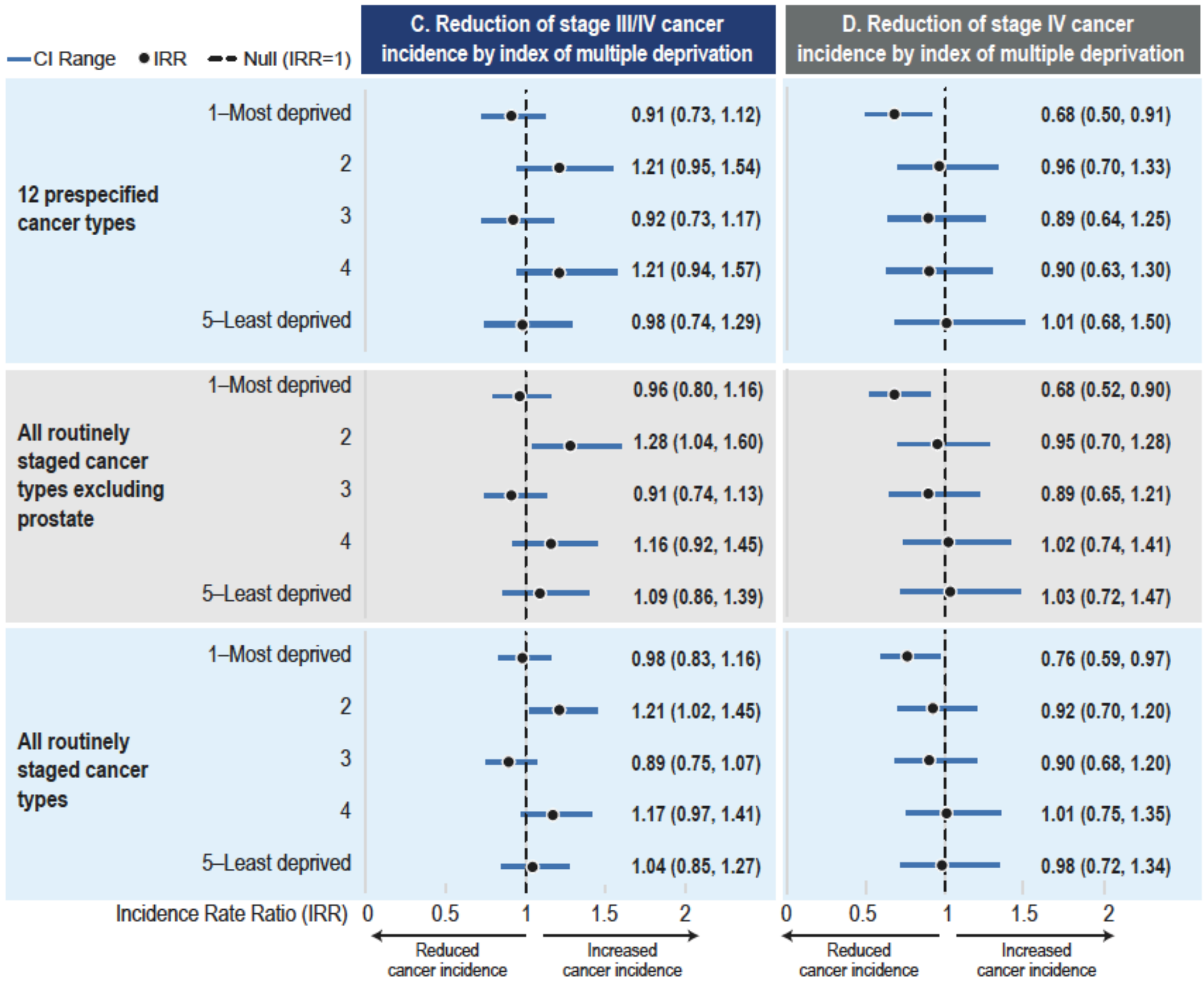
CI Range
IRR
Null (IRR=1)
C. Reduction of stage III/IV cancer incidence by index of multiple deprivation
D. Reduction of stage IV cancer incidence by index of multiple deprivation
12 prespecified cancer types
1–Most deprived
2
3
4
5–Least deprived
0.91 (0.73, 1.12)
1.21 (0.95, 1.54)
0.92 (0.73, 1.17)
1.21 (0.94, 1.57)
0.98 (0.74, 1.29)
0.68 (0.50, 0.91)
0.96 (0.70, 1.33)
0.89 (0.64, 1.25)
0.90 (0.63, 1.30)
1.01 (0.68, 1.50)
All routinely staged cancer types excluding prostate
1–Most deprived
2
3
4
5–Least deprived
0.96 (0.80, 1.16)
1.28 (1.04, 1.60)
0.91 (0.74, 1.13)
1.16 (0.92, 1.45)
1.09 (0.86, 1.39)
0.68 (0.52, 0.90)
0.95 (0.70, 1.28)
0.89 (0.65, 1.21)
1.02 (0.74, 1.41)
1.03 (0.72, 1.47)
All routinely staged cancer types
1–Most deprived
2
3
4
5–Least deprived
0.98 (0.83, 1.16)
1.21 (1.02, 1.45)
0.89 (0.75, 1.07)
1.17 (0.97, 1.41)
1.04 (0.85, 1.27)
0.76 (0.59, 0.97)
0.92 (0.70, 1.20)
0.90 (0.68, 1.20)
1.01 (0.75, 1.35)
0.98 (0.72, 1.34)
Incidence Rate Ratio (IRR)
0
0.5
1
1.5
2
Reduced cancer incidence
Increased cancer incidence

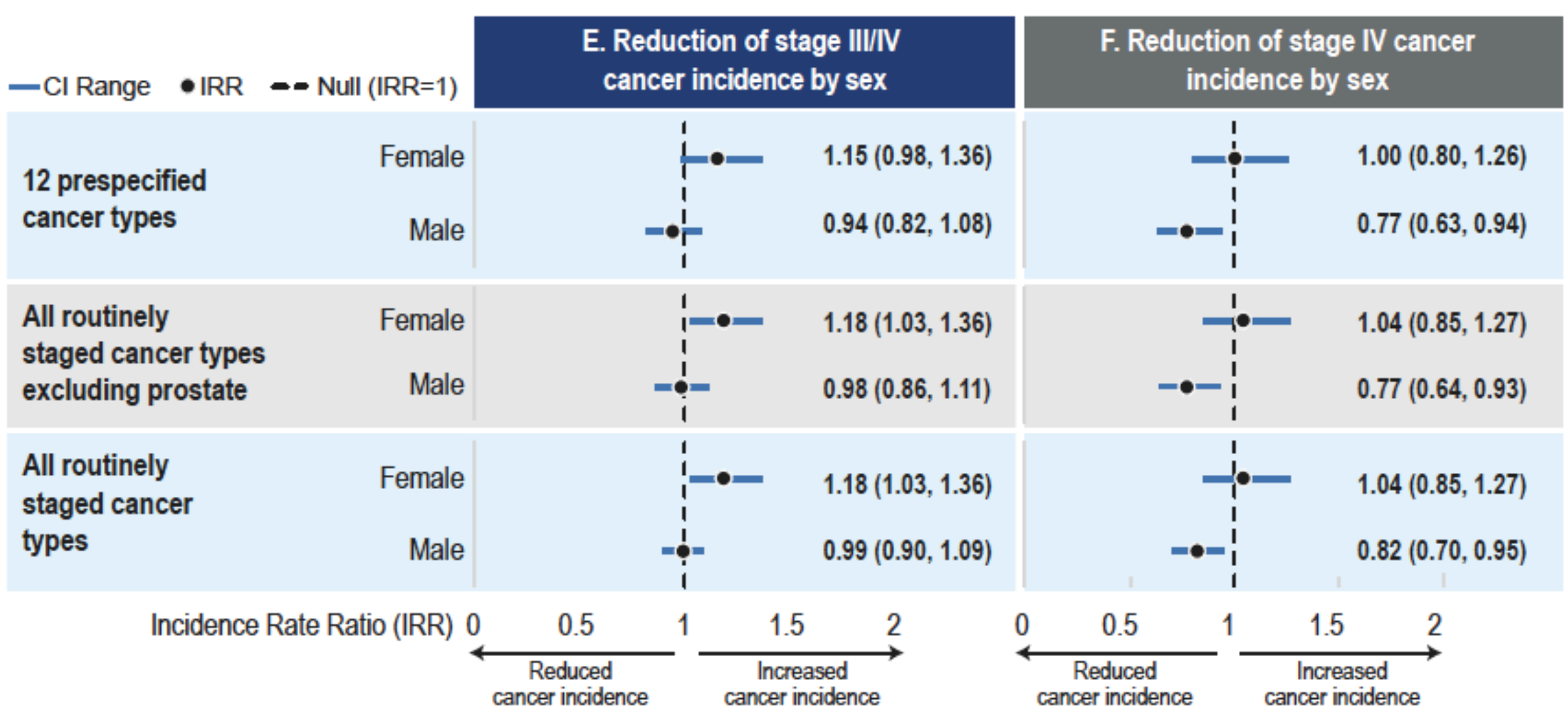
CI Range
IRR
Null (IRR=1)
E. Reduction of stage III/IV cancer incidence by sex
F. Reduction of stage IV cancer incidence by sex
12 prespecified cancer types
Female
Male
1.15 (0.98, 1.36)
0.94 (0.82, 1.08)
1.00 (0.80, 1.26)
0.77 (0.63, 0.94)
All routinely staged cancer types excluding prostate
Female
Male
1.18 (1.03, 1.36)
0.98 (0.86, 1.11)
1.04 (0.85, 1.27)
0.77 (0.64, 0.93)
All routinely staged cancer types
Female
Male
1.18 (1.03, 1.36)
0.99 (0.90, 1.09)
1.04 (0.85, 1.27)
0.82 (0.70, 0.95)
Incidence Rate Ratio (IRR)
0
0.5
1
1.5
2
Reduced cancer incidence
Increased cancer incidence

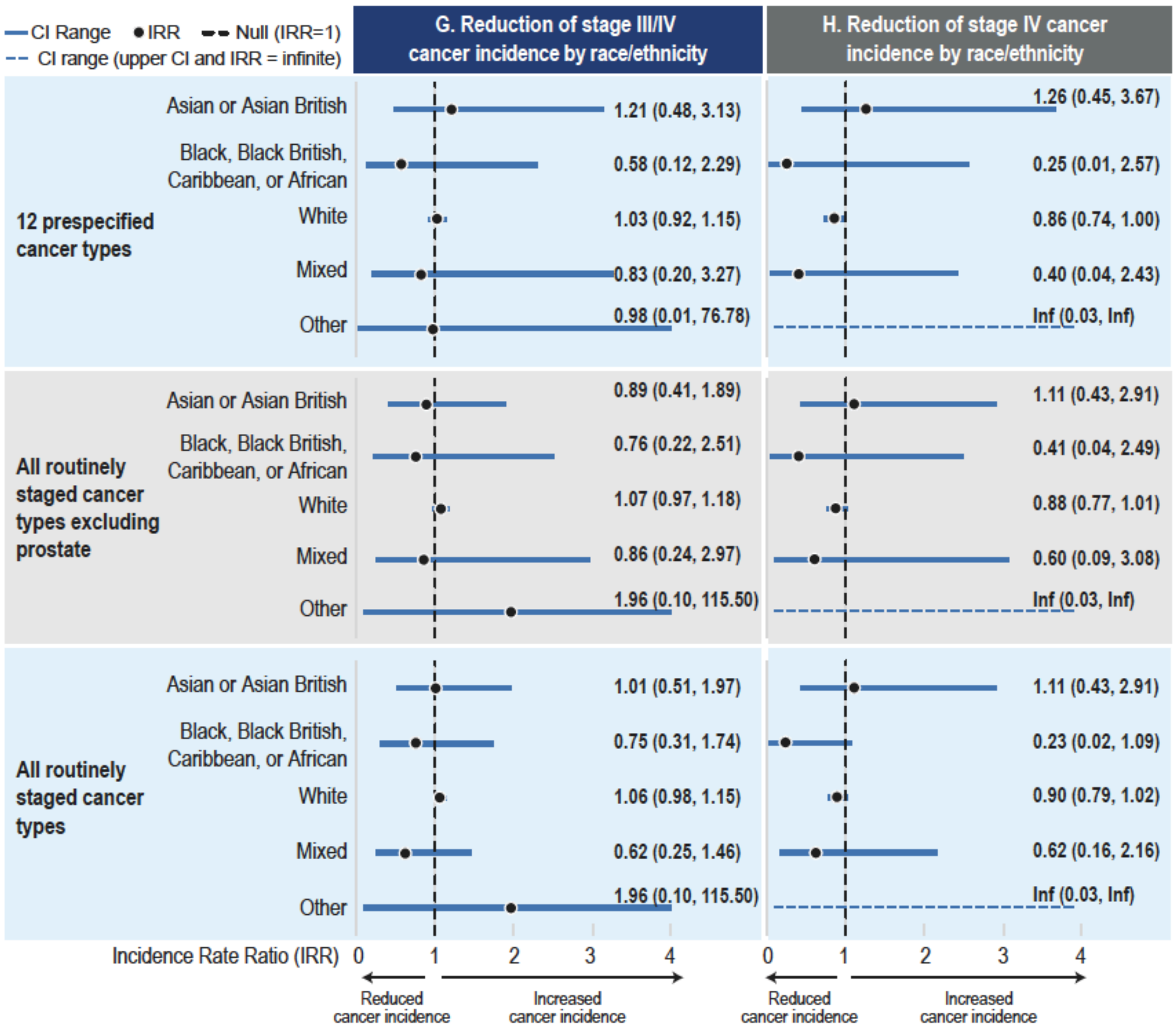
CI Range
IRR
Null (IRR=1)
CI range (upper CI and IRR = infinite)
G. Reduction of stage III/IV cancer incidence by race/ethnicity
H. Reduction of stage IV cancer incidence by race/ethnicity
12 prespecified cancer types
Asian or Asian British
Black, Black British, Caribbean, or African
White
Mixed
Other
1.21 (0.48, 3.13)
0.58 (0.12, 2.29)
1.03 (0.92, 1.15)
0.83 (0.20, 3.27)
0.98 (0.01, 76.78)
1.26 (0.45, 3.67)
0.25 (0.01, 2.57)
0.86 (0.74, 1.00)
0.40 (0.04, 2.43)
Inf (0.03, Inf)
All routinely staged cancer types excluding prostate
0.89 (0.41, 1.89)
0.76 (0.22, 2.51)
1.07 (0.97, 1.18)
0.86 (0.24, 2.97)
1.96 (0.10, 115.50)
1.11 (0.43, 2.91)
0.41 (0.04, 2.49)
0.88 (0.77, 1.01)
0.60 (0.09, 3.08)
Inf (0.03, Inf)
All routinely staged cancer types
1.01 (0.51, 1.97)
0.75 (0.31, 1.74)
1.06 (0.98, 1.15)
0.62 (0.25, 1.46)
1.96 (0.10, 115.50)
1.11 (0.43, 2.91)
0.23 (0.02, 1.09)
0.90 (0.79, 1.02)
0.62 (0.16, 2.16)
Inf (0.03, Inf)
Incidence Rate Ratio (IRR)
0 1 2 3 4
Reduced cancer incidence
Increased cancer incidence

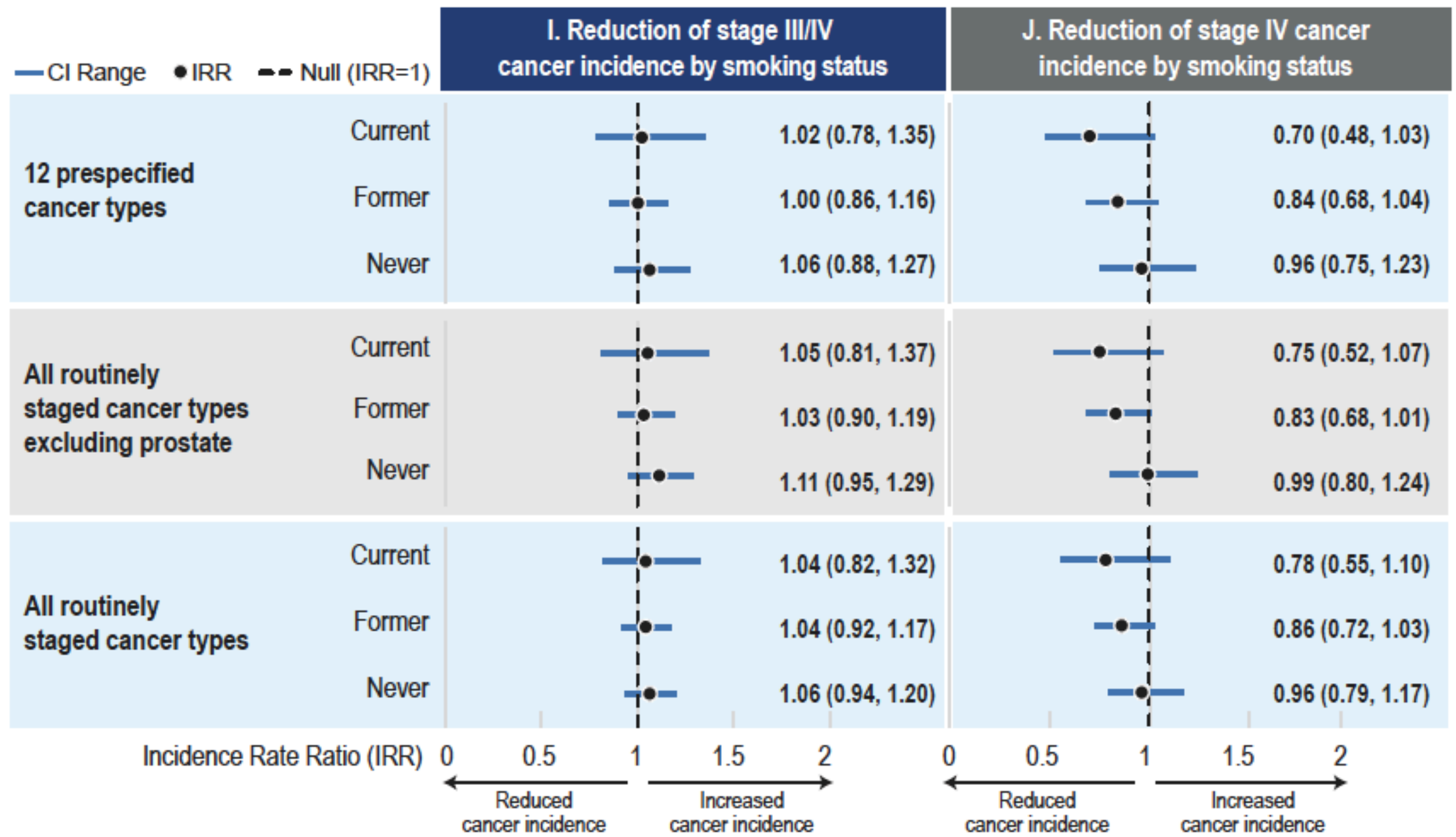
CI Range
IRR
Null (IRR=1)
I. Reduction of stage III/IV cancer incidence by smoking status
J. Reduction of stage IV cancer incidence by smoking status
12 prespecified cancer types
Current
Former
Never
All routinely staged cancer types excluding prostate
All routinely staged cancer types
1.02 (0.78, 1.35)
1.00 (0.86, 1.16)
1.06 (0.88, 1.27)
1.05 (0.81, 1.37)
1.03 (0.90, 1.19)
1.11 (0.95, 1.29)
1.04 (0.82, 1.32)
1.04 (0.92, 1.17)
1.06 (0.94, 1.20)
0.70 (0.48, 1.03)
0.84 (0.68, 1.04)
0.96 (0.75, 1.23)
0.75 (0.52, 1.07)
0.83 (0.68, 1.01)
0.99 (0.80, 1.24)
0.78 (0.55, 1.10)
0.86 (0.72, 1.03)
0.96 (0.79, 1.17)
Incidence Rate Ratio (IRR)
0
0.5
1
1.5
2
Reduced cancer incidence
Increased cancer incidence

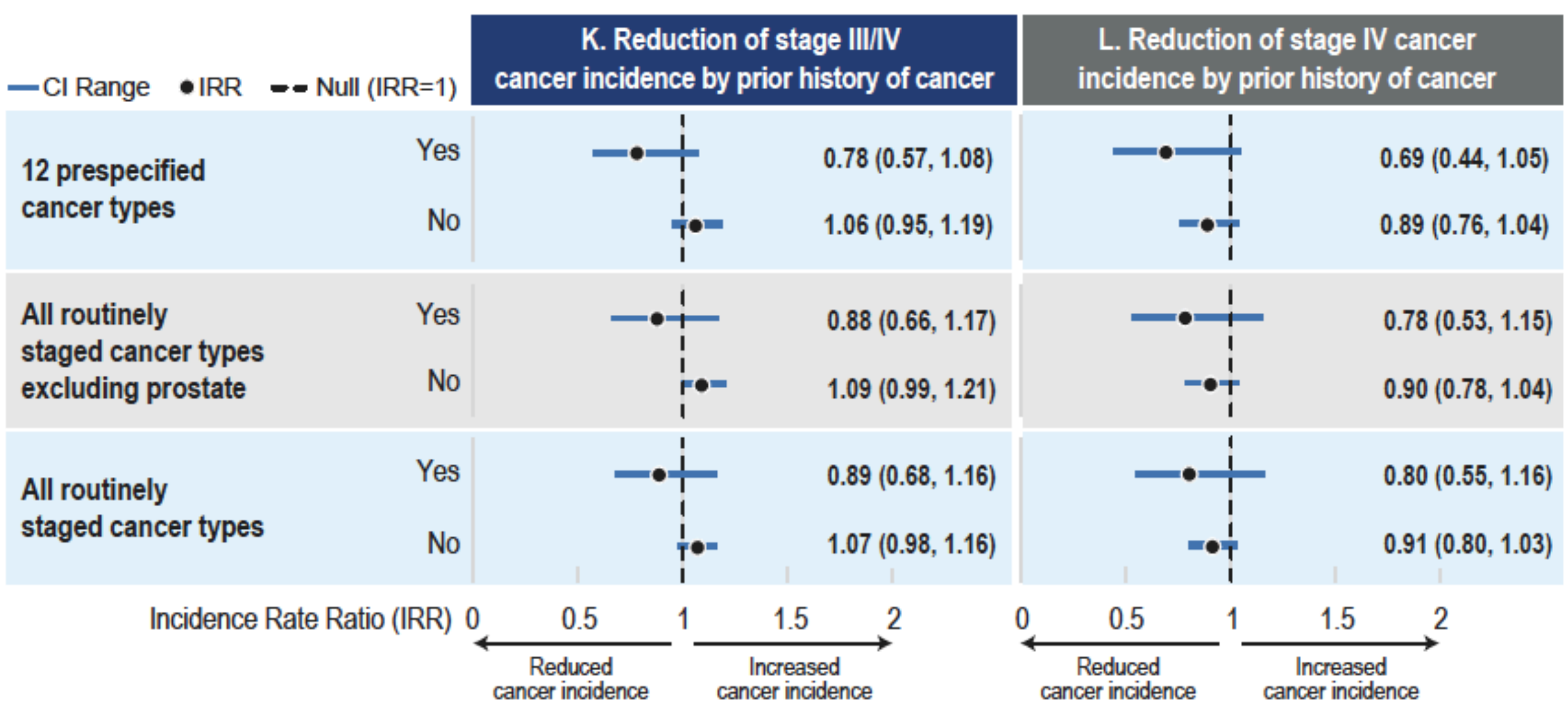
CI Range
IRR
Null (IRR=1)
K. Reduction of stage III/IV cancer incidence by prior history of cancer
L. Reduction of stage IV cancer incidence by prior history of cancer
12 prespecified cancer types
Yes
No
All routinely staged cancer types excluding prostate
All routinely staged cancer types
0.78 (0.57, 1.08)
1.06 (0.95, 1.19)
0.88 (0.66, 1.17)
1.09 (0.99, 1.21)
0.89 (0.68, 1.16)
1.07 (0.98, 1.16)
0.69 (0.44, 1.05)
0.89 (0.76, 1.04)
0.78 (0.53, 1.15)
0.90 (0.78, 1.04)
0.80 (0.55, 1.16)
0.91 (0.80, 1.03)
Incidence Rate Ratio (IRR)
0
0.5
1
1.5
2
Reduced cancer incidence
Increased cancer incidence

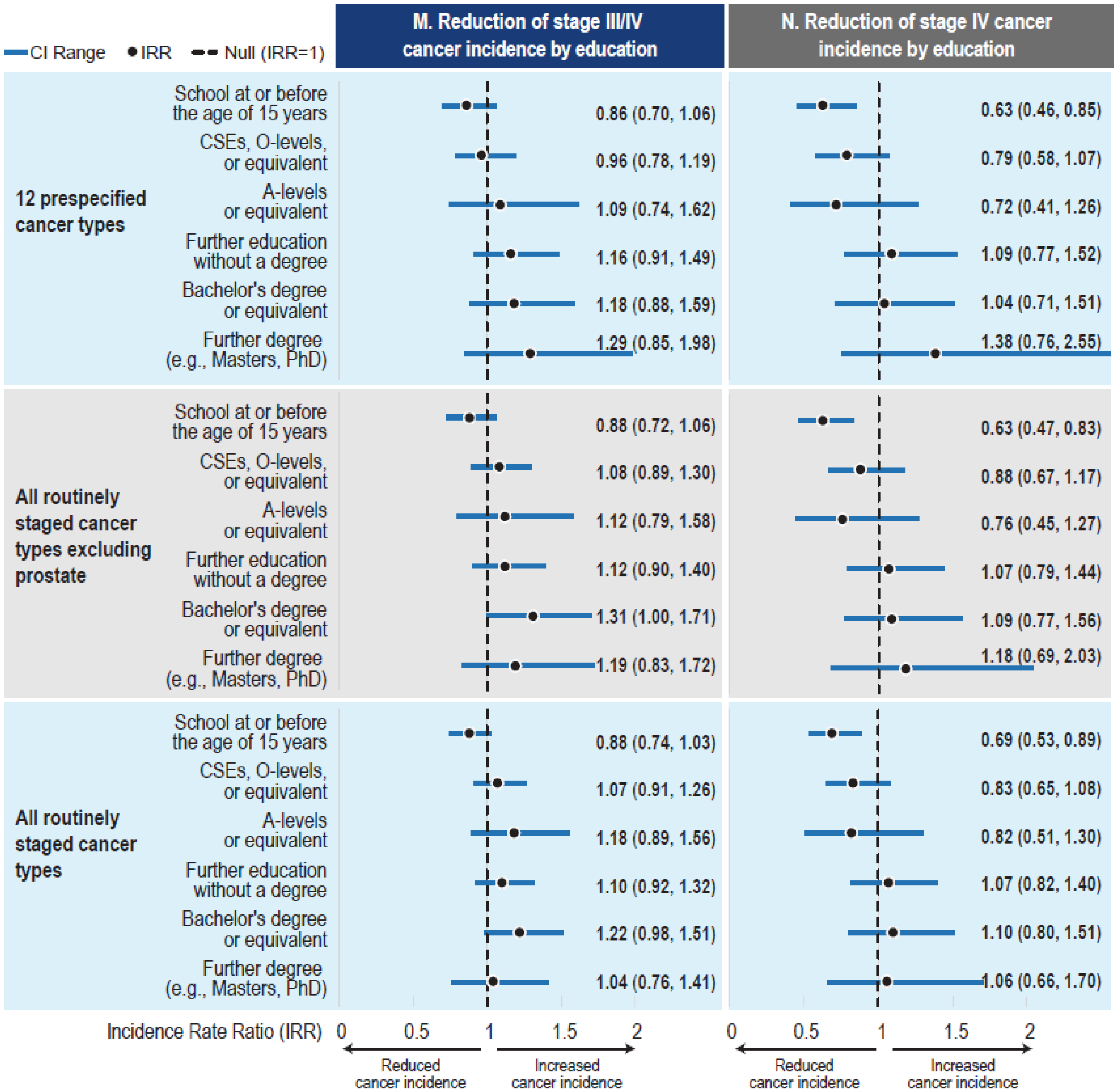
CI Range
IRR
Null (IRR=1)
M. Reduction of stage III/IV cancer incidence by education
N. Reduction of stage IV cancer incidence by education
12 prespecified cancer types
School at or before the age of 15 years
0.86 (0.70, 1.06)
0.63 (0.46, 0.85)
CSEs, O-levels, or equivalent
0.96 (0.78, 1.19)
0.79 (0.58, 1.07)
A-levels or equivalent
1.09 (0.74, 1.62)
0.72 (0.41, 1.26)
Further education without a degree
1.16 (0.91, 1.49)
1.09 (0.77, 1.52)
Bachelor's degree or equivalent
1.18 (0.88, 1.59)
1.04 (0.71, 1.51)
Further degree (e.g., Masters, PhD)
1.29 (0.85, 1.98)
1.38 (0.76, 2.55)
All routinely staged cancer types excluding prostate
School at or before the age of 15 years
0.88 (0.72, 1.06)
0.63 (0.47, 0.83)
CSEs, O-levels, or equivalent
1.08 (0.89, 1.30)
0.88 (0.67, 1.17)
A-levels or equivalent
1.12 (0.79, 1.58)
0.76 (0.45, 1.27)
Further education without a degree
1.12 (0.90, 1.40)
1.07 (0.79, 1.44)
Bachelor's degree or equivalent
1.31 (1.00, 1.71)
1.09 (0.77, 1.56)
Further degree (e.g., Masters, PhD)
1.19 (0.83, 1.72)
1.18 (0.69, 2.03)
All routinely staged cancer types
School at or before the age of 15 years
0.88 (0.74, 1.03)
0.69 (0.53, 0.89)
CSEs, O-levels, or equivalent
1.07 (0.91, 1.26)
0.83 (0.65, 1.08)
A-levels or equivalent
1.18 (0.89, 1.56)
0.82 (0.51, 1.30)
Further education without a degree
1.10 (0.92, 1.32)
1.07 (0.82, 1.40)
Bachelor's degree or equivalent
1.22 (0.98, 1.51)
1.10 (0.80, 1.51)
Further degree (e.g., Masters, PhD)
1.04 (0.76, 1.41)
1.06 (0.66, 1.70)
Incidence Rate Ratio (IRR)
0
0.5
1
1.5
2
Reduced cancer incidence
Increased cancer incidence
0
0.5
1
1.5
2
Reduced cancer incidence
Increased cancer incidence

Figure S4. Route to diagnosis distribution by arm in (A) prevalent screening round, (B-C) incident screening rounds, and (D) after 3 annual screening rounds.

The 'Other' category includes other outpatient, death certificate only, inpatient elective, unknown, and missing route to diagnosis.

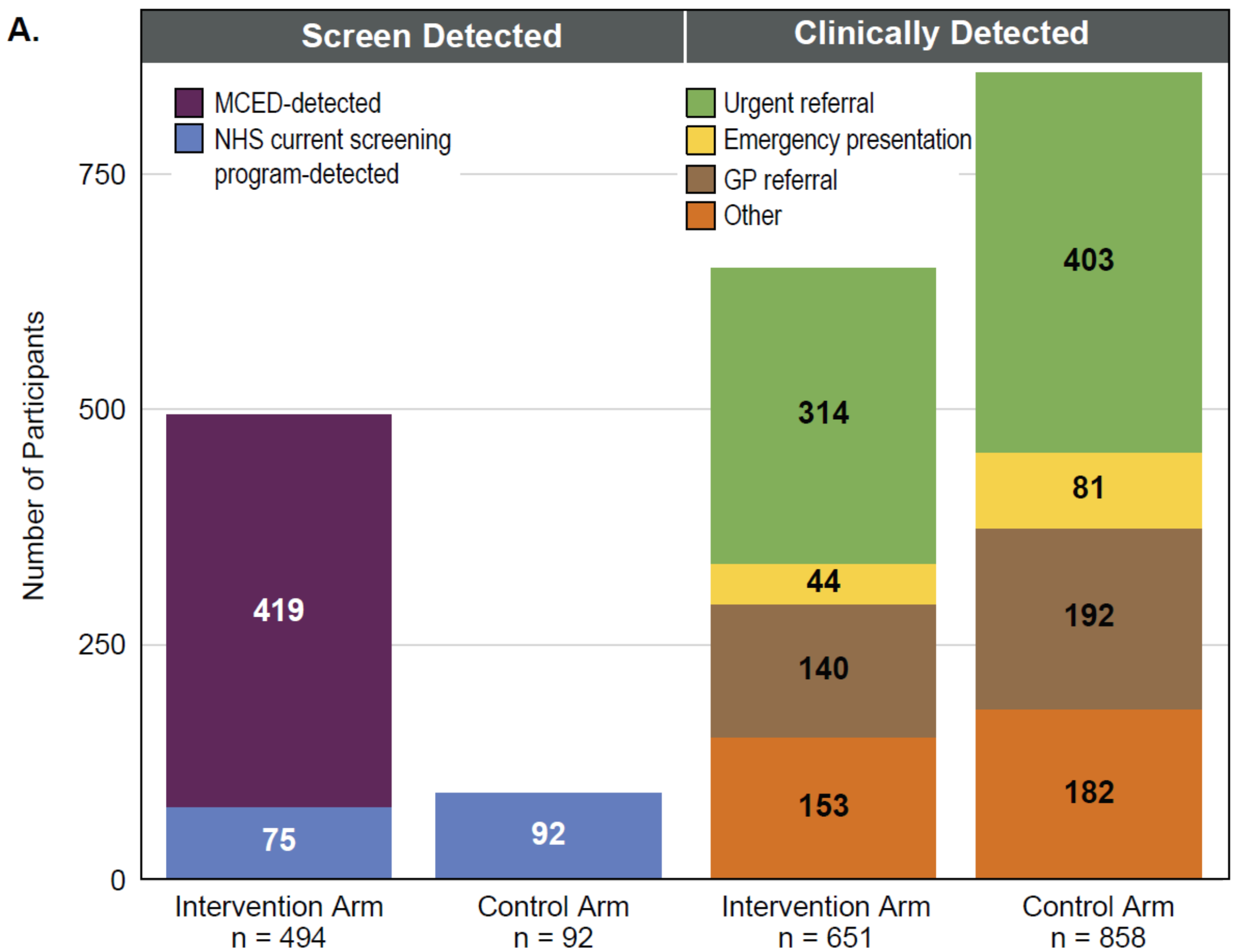

B.

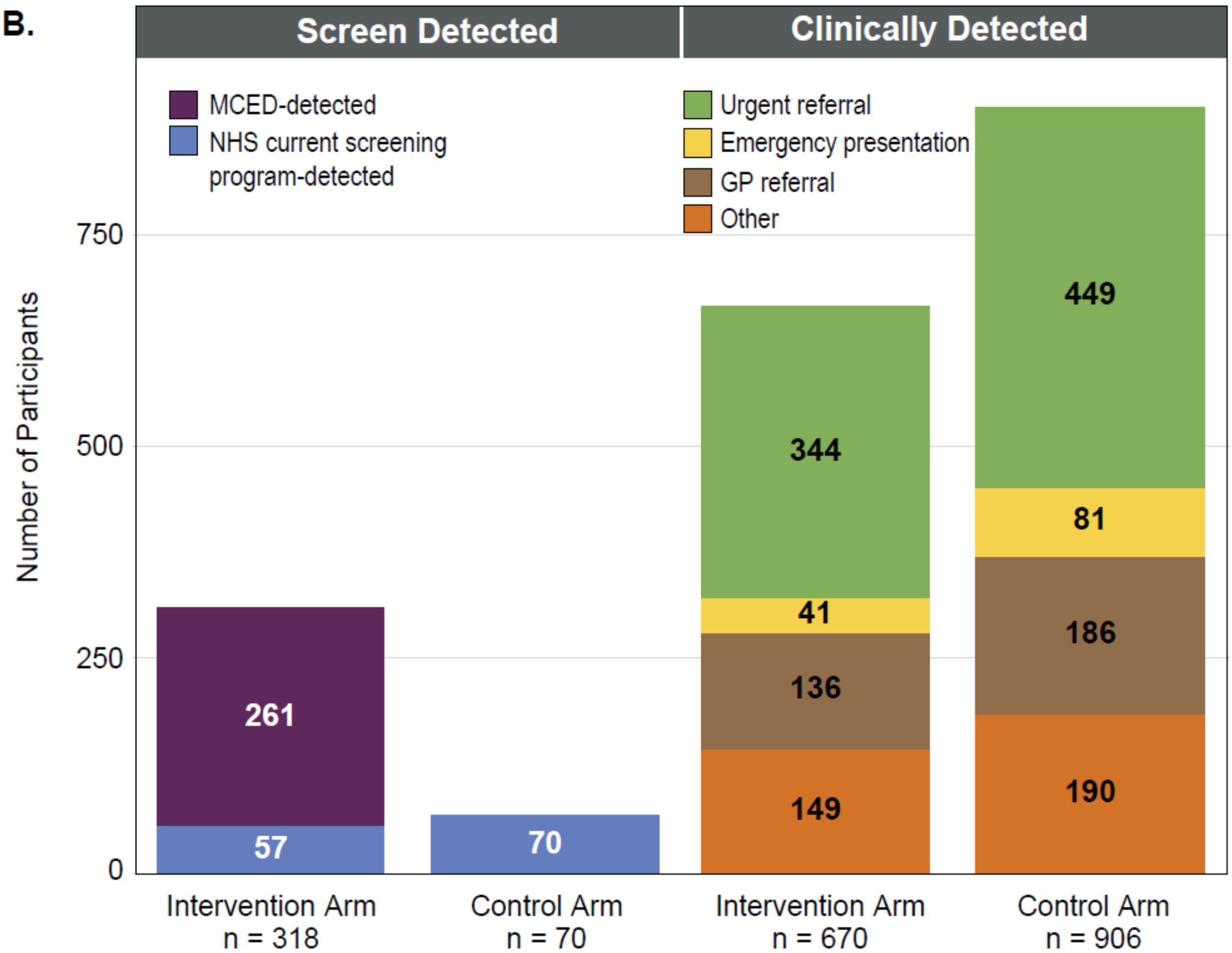

Screen Detected
Clinically Detected
MCED-detected
NHS current screening program-detected
Urgent referral
Emergency presentation
GP referral
Other
Number of Participants
750
500
250
0
261
57
70
344
41
136
149
449
81
186
190
Intervention Arm n = 318
Control Arm n = 70
Intervention Arm n = 670
Control Arm n = 906

C.

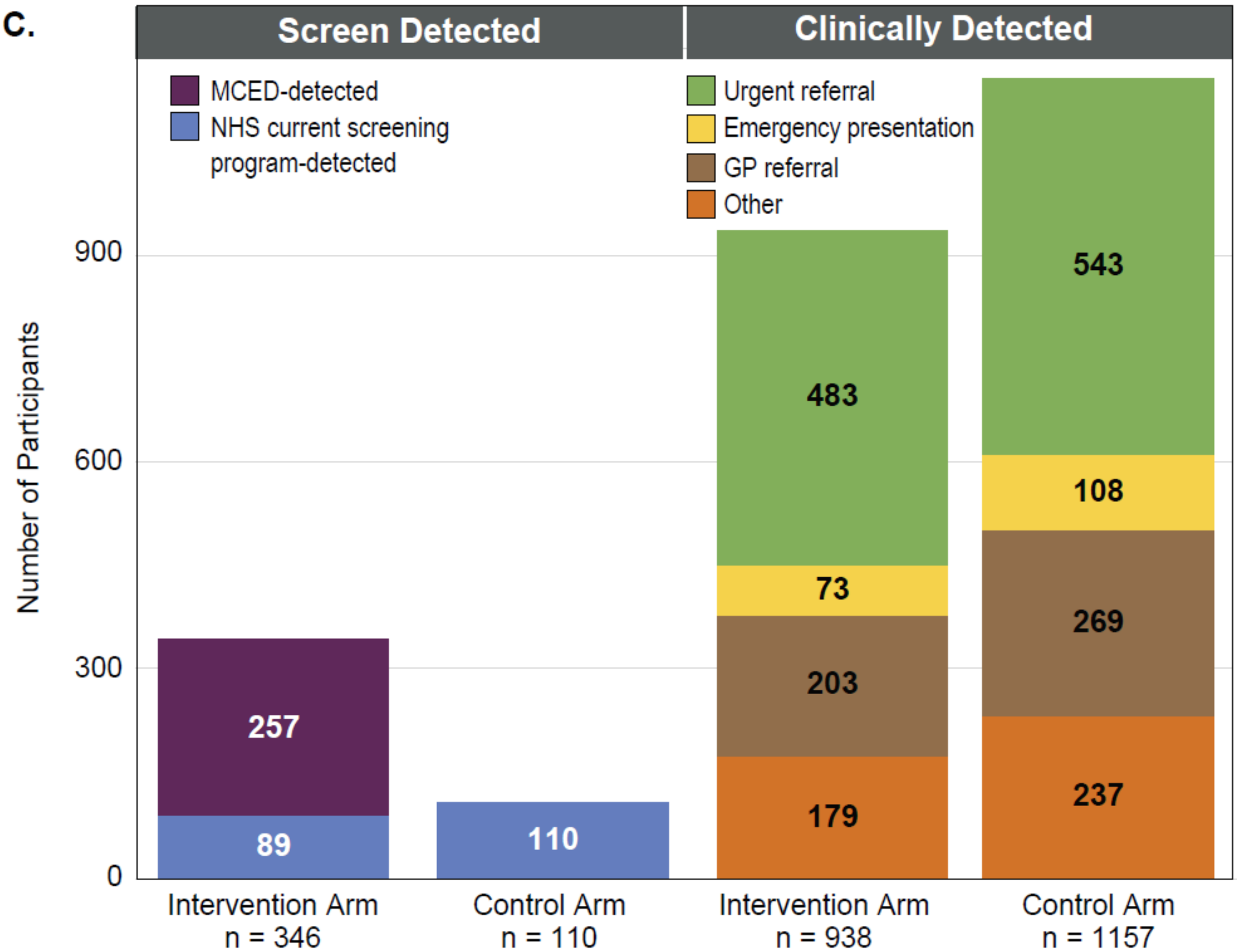

Screen Detected
Clinically Detected
MCED-detected
NHS current screening program-detected
Urgent referral
Emergency presentation
GP referral
Other
Number of Participants
900
600
300
0
257
89
110
483
73
203
179
543
108
269
237
Intervention Arm n = 346
Control Arm n = 110
Intervention Arm n = 938
Control Arm n = 1157

D.

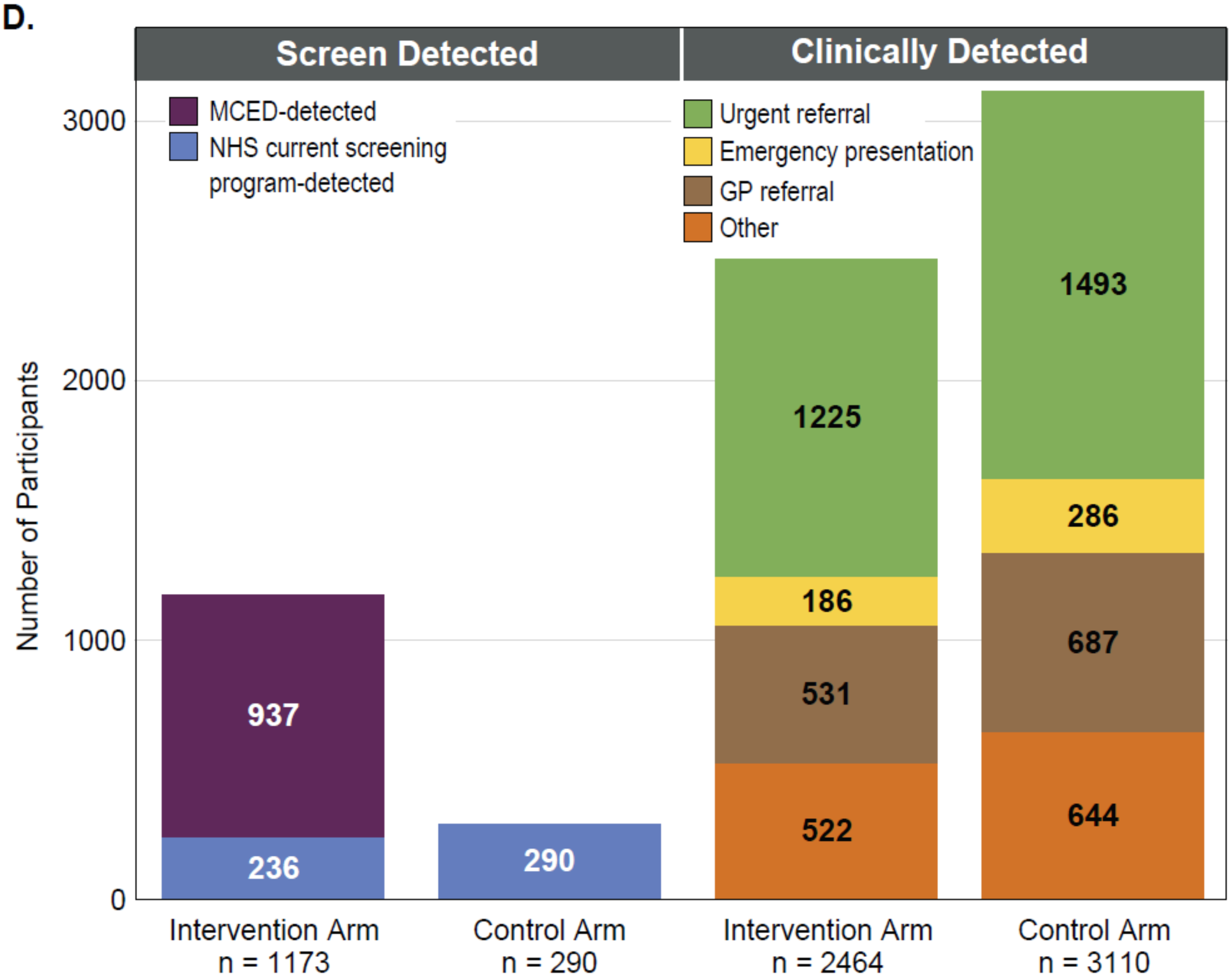
Screen Detected
Clinically Detected
MCED-detected
NHS current screening program-detected
Urgent referral
Emergency presentation
GP referral
Other
Number of Participants
3000
2000
1000
0
937
236
290
1225
186
531
522
1493
286
687
644
Intervention Arm n = 1173
Control Arm n = 290
Intervention Arm n = 2464
Control Arm n = 3110

Supplementary Tables

Table S1. Number of participants with at least one important protocol deviation by category and trial arm.

	Intervention arm (N = 71,122)	Control arm (N = 71,128)	Not randomized (N = 576)
**Total**	150 (0.21)	163 (0.23)	0 (0.00)
**Protocol Category**			
**Informed consent, n (%)**	142 (0.20)	155 (0.22)	0 (0.00)
**Eligibility criteria, n (%)**	3 (<0.01)	3 (<0.01)	0 (0.00)
**Good clinical practice, n (%)**	1 (<0.01)	0 (0.00)	0 (0.00)
**Safety, n (%)**	2 (<0.01)	4 (0.01)	0 (0.00)
**Procedure, n (%)**	2 (<0.01)	1 (<0.01)	0 (0.00)

Table S2. Number of important protocol deviations by category.

Category	Sub-category	Intervention arm (N = 71,122)	Control arm (N = 71,128)	Not randomized (N = 576)
**Informed consent (n)**	Impartial witness not present (reconsent required)	12	23	0
	Informed consent missing (missing completely, not conducted, reconsent required)	32	32	0
	Informed consent not signed by site staff (signature missing)	41	30	0

Category	Sub-category	Intervention arm (N = 71,122)	Control arm (N = 71,128)	Not randomized (N = 576)
	Participant has not marked (box is: blank) mandatory consent questions	57	70	0
**Eligibility criteria (n)**	Participant does not meet inclusion criteria #1. Participants must be at 50-77 years of age, inclusive, at the time of data extraction from NHS datasets or GP records used to identify potential participants	1	0	0

Category	Sub-category	Intervention arm (N = 71,122)	Control arm (N = 71,128)	Not randomized (N = 576)
	Participant failed eligibility exclusion criteria #4. Participant must not be currently taking demethylating or cytotoxic agents for any condition	1	3	0
	Participant failed eligibility exclusion criteria #5. Participant must not be undergoing current investigation for suspected cancer,	1	0	0

Category	Sub-category	Intervention arm (N = 71,122)	Control arm (N = 71,128)	Not randomized (N = 576)
	defined as having been referred to a two week wait clinic or undergoing investigations at an RDC or other clinic with a stated suspicion of cancer			
**Good clinical practice (n)**	Participant not withdrawn correctly / participant withdrawn incorrectly	1	0	0
**Safety (n)**	UADE or SAE not	2	4	0

Category	Sub-category	Intervention arm (N = 71,122)	Control arm (N = 71,128)	Not randomized (N = 576)
	reported within 24 hours			
**Procedure (n)**	Participant had blood drawn under incorrect participant ID in CRMS	1	1	0
	Sponsor SOP not followed	1	0	0

Table S3. Demographic characteristics of NHS-Galleri trial participants relative to England population.

		NHS-Galleri Randomized Analysis Set[a] (N = 142,250)	Cancer alliance regions[b,c]	England[b]
Age groups at randomization, n (%)	50-59 years	37,680 (26.5)	3,471,000 (42.7)	7,714,000 (43.2)
	60-69 years	57,388 (40.3)	2,767,000 (34.0)	6,023,000 (33.7)
	70-77 years	47,181 (33.2)[d]	1,910,000 (23.4)	4,120,000 (23.1)
Sex, n (%)	Female	71,462 (50.2)	4,176,000 (51.3)	9,169,000 (51.4)
	Male	70,788 (49.8)	3,972,000 (48.7)	8,687,000 (48.7)
Race/ethnicity, n (%)	Asian or Asian British	4725 (3.3)	415,000 (4.3)	1,127,000 (5.3)
	Black, Black British, Caribbean, or African	2054 (1.4)	236,000 (2.4)	594,000 (2.8)
	White	133,118 (93.6)	8,952,000 (91.8)	19,206,000 (89.9)
	Mixed[e]	1544 (1.1)	69,000 (0.7)	177,000 (0.8)
	Other[f]	627 (0.4)	80,000	267,000

		NHS-Galleri Randomized Analysis Set[a] (N = 142,250)	Cancer alliance regions[b,c]	England[b]
			(0.8)	(1.3)
	**Missing**	182 (0.1%)	NA	NA
**IMD quintile, n (%)**	**1 - Most deprived**	32,133 (22.6)	1,744,000 (21.4)	2,948,000 (16.7)
	**2**	27,922 (19.6)	1,562,000 (19.2)	3,285,000 (18.6)
	**3**	29,990 (21.1)	1,661,000 (20.4)	3,663,000 (20.8)
	**4**	28,848 (20.3)	1,655,000 (20.3)	3,837,000 (21.8)
	**5 - Least deprived**	22,883 (16.1)	1,527,000 (18.7)	3,894,000 (22.1)
	**Missing**	474 (0.3%)	NA	NA

IMD, index of multiple deprivation (an area-based measure of relative deprivation).

[a]Data originates from Table 1 of the manuscript

[b]Data originates from Swanton C, Bachtiar V, Mathews C, et al. NHS-Galleri trial: Enriched enrolment approaches and sociodemographic characteristics of enrolled participants. *Clin Trials*. 2025;22(2):227-238. doi:10.1177/17407745241302477.

[c]Northern, Cheshire and Merseyside, Greater Manchester, West Midlands, East Midlands, East of England (North), South East

London, and Kent and Medway.

[d]Individuals had to be aged 50-77 years at invitation; however, they could be aged 78 years by enrollment.

[e]Includes White and Black Caribbean; White and Black African; White and Asian; and any other Mixed or multiple ethnic background.

[f]Includes Arab and any other ethnic background not listed.

Table S4. NHS-Galleri trial retention in second and third screening round, by arm and demographic characteristics.

		Intervention arm			Control arm		
		First screening round (n)	Second screening round (n, %)	Third screening round (n, %)	First screening round (n)	Second screening round (n, %)	Third screening round (n, %)
**Total**		71,122	64,888	62,867	71,128	65,066	63,101
**Sex**	**Female**	35,723	32,680 (91.5)	31,705 (88.8)	35,739	32,785 (91.7)	31,885 (89.2)
	**Male**	35,399	32,208 (91.0)	31,162 (88.0)	35,389	32,281 (91.2)	31,216 (88.2)
**Age at enrollment**	**50-59 years**	18,791	16,678 (88.8)	16,240 (86.4)	18,889	16,885 (89.4)	16,379 (86.7)
	**60-69 years**	28,596	26,398 (92.3)	25,721 (89.9)	28,792	26,528 (92.1)	25,880 (89.9)

		Intervention arm			Control arm		
		First screening round (n)	Second screening round (n, %)	Third screening round (n, %)	First screening round (n)	Second screening round (n, %)	Third screening round (n, %)
	**70-79 years**	23,734	21,812 (91.9)	20,906 (88.1)	23,447	21,653 (92.3)	20,842 (88.9)
**Ethnicity[a]**	**Asian or Asian British**	2360	1968 (83.4)	1928 (81.7)	2365	1963 (83.0)	1948 (82.4)
	**Black, Black British, Caribbean, or African**	1016	811 (79.8)	804 (79.1)	1038	836 (80.5)	837 (80.6)

		Intervention arm			Control arm		
		First screening round (n)	Second screening round (n, %)	Third screening round (n, %)	First screening round (n)	Second screening round (n, %)	Third screening round (n, %)
	**White**	66,552	61,118 (91.8)	59,147 (88.9)	66,566	61,292 (92.1)	59,372 (89.2)
	**Mixed**[b]	776	647 (83.4)	650 (83.8)	768	652 (84.9)	633 (82.4)
	**Other**[c]	318	255 (80.2)	253 (79.6)	309	255 (82.5)	249 (80.6)

		Intervention arm			Control arm		
		First screening round (n)	Second screening round (n, %)	Third screening round (n, %)	First screening round (n)	Second screening round (n, %)	Third screening round (n, %)
Index of multiple deprivation (IMD) quintile[a]	1 - Most deprived	16,071	14,116 (87.8)	13,663 (85.0)	16,062	14,188 (88.3)	13,741 (85.5)
	2	13,942	12,594 (90.3)	12,150 (87.1)	13,980	12,695 (90.8)	12,260 (87.7)
	3	15,034	13,860 (92.2)	13,458 (89.5)	14,956	13,779 (92.1)	13,371 (89.4)
	4	14,372	13,368 (93.0)	12,964 (90.2)	14,476	13,503 (93.3)	13,101 (90.5)
	5 - Least deprived	11,469	10,747 (93.7)	10,427 (90.9)	11,414	10,680 (93.6)	10,413 (91.2)

[a]Participants with missing data for a demographic characteristic were omitted from the corresponding subgroup rows; subgroup totals may not sum to the total.

[b]Includes White and Black Caribbean; White and Black African; White and Asian; and any other Mixed or multiple ethnic background.

[c]Includes Arab and any other ethnic background not listed.

Table S5: Summary of all cancers diagnosed in the NHS-Galleri trial after 3 screening rounds.

	Intervention arm (N = 71,122)	Control arm (N = 71,128)
**Cancer type, n (%)**		
**Anus**	13 (0.02)	15 (0.02)
**Bladder**	54 (0.08)	68 (0.10)
**Breast**[a]	422 (0.59)	407 (0.57)
**Cervix**	8 (0.01)	5 (0.01)
**Colon/rectum**	337 (0.47)	280 (0.39)
**Gallbladder**	23 (0.03)	18 (0.03)
**Head and neck**	76 (0.11)	98 (0.14)
**Kidney**	75 (0.11)	60 (0.08)
**Liver/bile duct**	44 (0.06)	44 (0.06)
**Lung**	332 (0.47)	313 (0.44)
**Lymphoid leukemia**	68 (0.10)	58 (0.08)
**Lymphoma**	143 (0.20)	119 (0.17)

	Intervention arm (N = 71,122)	Control arm (N = 71,128)
**Melanoma**	181 (0.25)	183 (0.26)
**Myeloid neoplasm**	73 (0.10)	90 (0.13)
**Esophagus**	69 (0.10)	73 (0.10)
**Ovary**	62 (0.09)	39 (0.06)
**Pancreas**	97 (0.14)	86 (0.12)
**Plasma cell neoplasm**	81 (0.11)	48 (0.07)
**Prostate**	974 (1.37)	918 (1.29)
**Sarcoma**	29 (0.04)	29 (0.04)
**Stomach**	32 (0.05)	27 (0.04)
**Thyroid**	25 (0.04)	27 (0.04)
**Urothelial tract**	15 (0.02)	17 (0.02)
**Uterus**	96 (0.14)	73 (0.10)
**Other**[b]	124 (0.17)	106 (0.15)
**Multiple**	166 (0.23)	173 (0.24)

	Intervention arm (N = 71,122)	Control arm (N = 71,128)
**NCRAS cancer of unknown primary**	18 (0.03)	26 (0.04)
**Cancer stage per the highest routinely staged cancer, n (%)**		
**Total routinely staged[a]**	3431 (4.82)	3177 (4.47)
**Stage I**	1383 (1.95)	1308 (1.84)
**Stage II**	673 (0.95)	574 (0.81)
**Stage III**	763 (1.07)	645 (0.91)
**Stage IV**	503 (0.71)	563 (0.79)
**Missing**	107 (0.15)	85 (0.12)
**Not routinely staged / Unknown**	206 (0.29)	223 (0.31)
**Route to cancer diagnosis[b]**		
**Screening**	265 (0.37%)	290 (0.41%)

	Intervention arm (N = 71,122)	Control arm (N = 71,128)
**Urgent cancer referral (Two Week Wait)**	1775 (2.50)	1493 (2.10)
**Emergency presentation**[c]	225 (0.32)	286 (0.40)
**GP referral**	674 (0.95)	687 (0.97)
**Other outpatient**	325 (0.46)	272 (0.38)
**Inpatient elective**	37 (0.05)	40 (0.06)
**Death certificate only**	11 (0.02)	16 (0.02)
**Unknown**	57 (0.08)	60 (0.08)
**Missing**	268 (0.38)	256 (0.36)

[a]There were 2 participants with stage 0 cancer in each of the intervention and control arms. All of these were Paget's disease, which is invasive at stage 0.

[b]Based on the earliest diagnosed cancer if a participant had multiple cancers. MCED test-positive participants are referred through the urgent cancer referral pathway (Two Week Wait).

[c]The intention-to-treat randomized comparison using NCRAS-provided route to diagnosis data, whereby 225 participants in the intervention arm and 286 participants in the control arm had an

emergency presentation route to diagnosis, corresponding to an observed 21% reduction in emergency presentations in the intervention arm. Following identification of some data discrepancies, an independent targeted review by an NCRAS analyst of 39 participants with a positive MCED test result who were diagnosed via emergency presentation found that 8 had evidence of referral via an urgent cancer referral pathway, 2 via a routine GP referral and 2 had an unknown route; the remaining 27 were still classified as emergency presentations. Reclassifying the 8 participants with a positive MCED test result who had evidence of an urgent cancer referral, plus the 2 with routine GP referral and 2 with unknown route from emergency to non-emergency presentation, would reduce the intervention arm emergency presentation count from 225 to 213 (vs 286 in the control arm), giving a 25.5% lower count in the intervention arm relative to the control arm. No control-arm participants were reviewed.

Table S6: Distribution of routinely staged cancers by cancer type and stage in the NHS-Galleri trial after 3 screening rounds.

	Intervention arm						Control arm					
Cancer type[a]	Stage I n (%)	Stage II n (%)	Stage III n (%)	Stage IV n (%)[b]	Missing n (%)[c]	Total n (% of all cancer types)[d]	Stage I n (%)	Stage II n (%)	Stage III n (%)	Stage IV n (%)[b]	Missing n (%)[c]	Total n (% of all cancer types)[d]
**Total**	1383 (40.3)	673 (19.6)	763 (22.2)	503 (14.7)	107 (3.1%)	3431	1308 (41.2)	574 (18.1)	645 (20.3)	563 (17.7)	85 (2.7)	3177
**Anus**	5 (38.5)	1 (7.7)	6 (46.2)	1 (7.7)	0	13 (0.4)	3 (21.4)	6 (42.9)	5 (35.7)	0	0	14 (0.4)
**Bladder**	34 (56.7)	14 (23.3)	8 (13.3)	3 (5.0)	1 (1.7)	60 (1.7)	36 (47.4)	14 (18.4)	19 (25.0)	6 (7.9)	1 (1.3)	76 (2.4)
**Breast**	211 (47.5)	185 (41.7)	28 (6.3)	16 (3.6)	3 (0.7)	444 (12.9)	227 (53.5)	159 (37.5)	24 (5.7)	10 (2.4)	2 (0.5)	424 (13.3)
**Cervix**	3 (37.5)	3 (37.5)	1 (12.5)	1 (12.5)	0	8 (0.2)	2 (40.0)	2 (40.0)	0	1 (20.0)	0	5 (0.2)
**Colon/rectum**	136 (38.3)	74 (20.8)	98 (27.6)	45 (12.7)	2 (0.6)	355 (10.3)	86 (27.7)	77 (24.8)	96 (31.0)	49 (15.8)	2 (0.6)	310 (9.8)

Cancer type[a]	Intervention arm						Control arm					
	Stage I n (%)	Stage II n (%)	Stage III n (%)	Stage IV n (%)[b]	Missing n (%)[c]	Total n (% of all cancer types)[d]	Stage I n (%)	Stage II n (%)	Stage III n (%)	Stage IV n (%)[b]	Missing n (%)[c]	Total n (% of all cancer types)[d]
**Gallbladder**	1 (4.0)	7 (28.0)	5 (20.0)	11 (44.0)	1 (4.0)	25 (0.7)	2 (10.0)	8 (40.0)	3 (15.0)	7 (35.0)	0	20 (0.6)
**Head and neck**	39 (48.1)	10 (12.3)	17 (21.0)	15 (18.5)	0	81 (2.4)	42 (41.6)	15 (14.9)	18 (17.8)	24 (23.8)	2 (2.0)	101 (3.2)
**Kidney**	38 (44.7)	8 (9.4)	25 (29.4)	12 (14.1)	2 (2.4)	85 (2.5)	39 (55.7)	3 (4.3)	17 (24.3)	9 (12.9)	2 (2.9)	70 (2.2)
**Liver/bile duct**	14 (29.2)	16 (33.3)	7 (14.6)	9 (18.8)	2 (4.2)	48 (1.4)	12 (27.3)	10 (22.7)	4 (9.1)	16 (36.4)	2 (4.5)	44 (1.4)
**Lung**	116 (33.0)	35 (9.9)	101 (28.7)	99 (28.1)	1 (0.3)	352 (10.3)	129 (37.7)	28 (8.2)	58 (17.0)	123 (36.0)	4 (1.2)	342 (10.8)
**Lymphoma**	31 (20.3)	16 (10.5)	42 (27.5)	62 (40.5)	2 (1.3)	153 (4.5)	26 (20.3)	10 (7.8)	25 (19.5)	66 (51.6)	1 (0.8)	128 (4.0)
**Melanoma**	127 (66.8)	40 (21.1)	17 (8.9)	3 (1.6)	3 (1.6)	190 (5.5)	146 (75.6)	30 (15.5)	12 (6.2)	4 (2.1)	1 (0.5)	193 (6.1)

Cancer type[a]	Intervention arm Stage I n (%)	Stage II n (%)	Stage III n (%)	Stage IV n (%)[b]	Missing n (%)[c]	Total n (% of all cancer types)[d]	Control arm Stage I n (%)	Stage II n (%)	Stage III n (%)	Stage IV n (%)[b]	Missing n (%)[c]	Total n (% of all cancer types)[d]
**Esophagus**	8 (10.7)	17 (22.7)	26 (34.7)	19 (25.3)	5 (6.7)	75 (2.2)	3 (3.8)	10 (12.7)	24 (30.4)	38 (48.1)	4 (5.1)	79 (2.5)
**Ovary**	13 (19.7)	5 (7.6)	34 (51.5)	14 (21.2)	0	66 (1.9)	7 (17.5)	2 (5.0)	18 (45.0)	12 (30.0)	1 (2.5)	40 (1.3)
**Pancreas**	10 (10.0)	18 (18.0)	12 (12.0)	59 (59.0)	1 (1.0)	100 (2.9)	15 (17.2)	8 (9.2)	19 (21.8)	43 (49.4)	2 (2.3)	87 (2.7)
**Plasma cell neoplasm**	13 (15.3)	11 (12.9)	1 (1.2)	2 (2.4)	58 (68.2)	85 (2.5)	7 (14.0)	4 (8.0)	0	1 (2.0)	38 (76.0)	50 (1.6)
**Prostate**	445 (44.5)	179 (17.9)	274 (27.4)	93 (9.3)	9 (0.9)	1000 (29.1)	411 (43.3)	159 (16.8)	269 (28.3)	99 (10.4)	11 (1.2)	949 (29.9)
**Sarcoma**	9 (33.3)	3 (11.1)	8 (29.6)	1 (3.7)	6 (22.2)	27 (0.8)	3 (11.1)	8 (29.6)	6 (22.2)	4 (14.8)	6 (22.2)	27 (0.8)
**Stomach**	2 (6.1)	9 (27.3)	8 (24.2)	13 (39.4)	1 (3.0)	33 (1.0)	4 (14.8)	5 (18.5)	1 (3.7)	16 (59.3)	1 (3.7)	27 (0.8)

Cancer type[a]	Intervention arm						Control arm					
	Stage I n (%)	Stage II n (%)	Stage III n (%)	Stage IV n (%)[b]	Missing n (%)[c]	Total n (% of all cancer types)[d]	Stage I n (%)	Stage II n (%)	Stage III n (%)	Stage IV n (%)[b]	Missing n (%)[c]	Total n (% of all cancer types)[d]
**Thyroid**	15 (57.7)	7 (26.9)	0	4 (15.4)	0	26 (0.8)	17 (63.0)	4 (14.8)	2 (7.4)	3 (11.1)	1 (3.7)	27 (0.8)
**Urothelial tract**	3 (17.6)	2 (11.8)	8 (47.1)	3 (17.6)	1 (5.9)	17 (0.5)	2 (9.5)	3 (14.3)	7 (33.3)	9 (42.9)	0	21 (0.7)
**Uterus**	75 (75.8)	5 (5.1)	10 (10.1)	8 (8.1)	1 (1.0)	99 (2.9)	59 (81.9)	4 (5.6)	4 (5.6)	4 (5.6)	1 (1.4)	72 (2.3)
**Other**	32 (38.6)	6 (7.2)	26 (31.3)	10 (12.0)	8 (9.6)	83 (2.4)	29 (41.4)	5 (7.1)	14 (20.0)	19 (27.1)	3 (4.3)	70 (2.2)
**Multiple**	3 (50.0)	2 (33.3)	1 (16.7)	0	0	6 (0.2)	1 (100.0)	0	0	0	0	1 (<0.1)

[a]Cancer type per the highest-staged cancer. If there are multiple cancers with the same highest stage, the earliest diagnosed cancer is selected.

[b]For ISS the stage III represents the most advanced stage in the staging system, thus it is mapped to the most advanced stage for the routinely staged cancers.

[c]Missing includes those cancers with insufficient information/not staged.

[d]There were 2 participants with stage 0 cancer in each of the intervention and control arms. All of these were Paget's disease, which is invasive at stage 0.

Table S7. Distribution of routinely staged cancers by stage across individual screening rounds.

	Total		First (prevalent) screening round[a]		Second (incident) screening round[a]		Third (incident) screening round with primary study follow-up window[a]	
	Intervention arm	Control arm	Intervention arm	Control arm	Intervention arm	Control arm	Intervention arm	Control arm
**12 prespecified cancer types[b]**								
**Cancer participants**	1421	1298	469	383	373	363	477	471
**Stage I, n (%)**	421 (29.6)	370 (28.5)	119 (25.4)	100 (26.1)	124 (33.2)	112 (30.9)	157 (32.9)	136 (28.9)
**Stage II, n (%)**	226 (15.9)	189 (14.6)	76 (16.2)	52 (13.6)	56 (15.0)	51 (14.0)	79 (16.6)	74 (15.7)

	Total		First (prevalent) screening round[a]		Second (incident) screening round[a]		Third (incident) screening round with primary study follow-up window[a]	
	Intervention arm	Control arm	Intervention arm	Control arm	Intervention arm	Control arm	Intervention arm	Control arm
**Stage III, n (%)**	360 (25.3)	287 (22.1)	133 (28.4)	83 (21.7)	100 (26.8)	88 (24.2)	108 (22.6)	99 (21.0)
**Stage IV, n (%)**	341 (24.0)	394 (30.4)	117 (24.9)	127 (33.2)	77 (20.6)	98 (27.0)	104 (21.8)	142 (30.1)
**Missing, n (%)**	73 (5.1)	58 (4.5)	24 (5.1)	21 (5.5)	16 (4.3)	14 (3.9)	29 (6.1)	20 (4.2)
**All routinely staged cancer types excluding prostate**								
**Cancer participants[c]**	2431	2228	786	658	639	610	851	815

	Total		First (prevalent) screening round[a]		Second (incident) screening round[a]		Third (incident) screening round with primary study follow-up window[a]	
	**Intervention arm**	**Control arm**	**Intervention arm**	**Control arm**	**Intervention arm**	**Control arm**	**Intervention arm**	**Control arm**
**Stage I, n (%)**	938 (38.6)	897 (40.3)	277 (35.2)	256 (38.9)	259 (40.5)	259 (42.5)	357 (42.0)	333 (40.9)
**Stage II, n (%)**	494 (20.3)	415 (18.6)	161 (20.5)	118 (17.9)	133 (20.8)	106 (17.4)	175 (20.6)	162 (19.9)
**Stage III, n (%)**	489 (20.1)	376 (16.9)	177 (22.5)	106 (16.1)	133 (20.8)	110 (18.0)	149 (17.5)	131 (16.1)
**Stage IV, n (%)**	410 (16.9)	464 (20.8)	137 (17.4)	149 (22.6)	94 (14.7)	115 (18.9)	130 (15.3)	165 (20.2)

	Total		First (prevalent) screening round[a]		Second (incident) screening round[a]		Third (incident) screening round with primary study follow-up window[a]	
	Intervention arm	Control arm	Intervention arm	Control arm	Intervention arm	Control arm	Intervention arm	Control arm
**Missing, n (%)**	98 (4.0)	74 (3.3)	34 (4.3)	28 (4.3)	19 (3.0)	19 (3.1)	40 (4.7)	24 (2.9)
**All routinely staged cancer types**								
**Cancer participants[c]**	3431	3177	1078	898	933	901	1208	1182
**Stage I, n (%)**	1383 (40.3)	1308 (41.2)	415 (38.5)	366 (40.8)	387 (41.5)	384 (42.6)	516 (42.7)	487 (41.2)
**Stage II, n (%)**	673 (19.6)	574 (18.1)	212 (19.7)	159 (17.7)	178 (19.1)	159 (17.6)	243 (20.1)	221 (18.7)

	Total		First (prevalent) screening round[a]		Second (incident) screening round[a]		Third (incident) screening round with primary study follow-up window[a]	
	Intervention arm	Control arm	Intervention arm	Control arm	Intervention arm	Control arm	Intervention arm	Control arm
**Stage III, n (%)**	763 (22.2)	645 (20.3)	249 (23.1)	172 (19.2)	225 (24.1)	183 (20.3)	248 (20.5)	246 (20.8)
**Stage IV, n (%)**	503 (14.7)	563 (17.7)	164 (15.2)	169 (18.8)	120 (12.9)	154 (17.1)	158 (13.1)	198 (16.8)
**Missing, n (%)**	107 (3.1)	85 (2.7)	38 (3.5)	31 (3.5)	22 (2.4)	20 (2.2)	43 (3.6)	30 (2.5)

[a]The first round of screening was from the date of the first blood draw in that round to the date of the first blood draw in the second round; the second round was from the date of the first blood draw in the second round to the date of the first blood draw in the third round; and the third round was from the date of the first blood draw in the third round to the end of follow-up on 12 July 2025.

[b]Lung, head and neck, colon/rectum, pancreas, myeloma/plasma cell neoplasm, liver/bile duct, stomach, esophagus, anus, lymphoma, ovary, and bladder.

[c]There were 2 participants with stage 0 cancer in each of the intervention and control arms. All of these were Paget's disease, which is invasive at stage 0.

Note: All results for 12 prespecified cancer types, all results for all routinely staged cancer types excluding prostate, as well as the results in the "total" columns for all routinely staged cancer types presented in this table are from post-hoc analyses. The apparent discrepancy in the number of stage IV cancers of the 12 prespecified types between Table 2 and Table S6 reflects how participants with multiple primary cancers are counted. As prespecified in the SAP, Table 2 counts participants with a qualifying stage IV cancer of one of the 12 prespecified types during follow-up. In contrast, Table S6 serves as a participant-level descriptive summary, assigning each participant a single cancer/stage based on the highest stage, with ties resolved by the earliest diagnosis. Therefore, participants with an earlier stage IV cancer outside the 12 prespecified types and a subsequent stage IV cancer of one of the 12 prespecified types are counted in Table 2 but not in the stage IV 12-prespecified-type category in Table S6.

Table S8. Reduction in stage III/IV cancers after each annual screening round in the NHS-Galleri trial.

	Prespecified group of 12 cancer types[a]		All routinely staged cancer types excluding prostate cancer		All routinely staged cancer types	
	Intervention arm	Control arm	Intervention arm	Control arm	Intervention arm	Control arm
**First (prevalent) screening round**						
**Participants (n)**	71,122	71,128	71,122	71,128	71,122	71,128
**Participants with stage III/IV cancer, n (%)**	250 (0.35)	211 (0.30)	314 (0.44)	256 (0.36)	413 (0.58)	341 (0.48)
**Follow-up, person-years**	70,161	70,221	70,130	70,203	70,076	70,166

	Prespecified group of 12 cancer types[a]		All routinely staged cancer types excluding prostate cancer		All routinely staged cancer types	
	Intervention arm	Control arm	Intervention arm	Control arm	Intervention arm	Control arm
**Incidence rate per 100,000 person-years (95% CI)**	356.3 (313.5, 403.3)	300.5 (261.3, 343.9)	447.7 (399.6, 500.1)	364.7 (321.3, 412.2)	589.4 (533.9, 649.0)	486.0 (435.8, 540.4)
**Incidence rate difference (95% CI)**	55.8 (−4.1, 116.0)		83.1 (16.4, 150.0)		103.4 (26.7, 180.4)	
**Incidence rate ratio (95% CI)**	1.19 (0.98, 1.43)		1.23 (1.04, 1.45)		1.21 (1.05, 1.40)	
**Second (incident) screening round**						

	Prespecified group of 12 cancer types[a]		All routinely staged cancer types excluding prostate cancer		All routinely staged cancer types	
	Intervention arm	Control arm	Intervention arm	Control arm	Intervention arm	Control arm
**Participants (n)**	64,888	65,066	64,888	65,066	64,888	65,066
**Participants with stage III/IV cancer, n (%)**	179 (0.28)	189 (0.29)	229 (0.35)	228 (0.35)	345 (0.53)	337 (0.52)
**Follow-up, person-years**	65,601	65,815	65,575	65,799	65,516	65,748
**Incidence rate per 100,000 person-years (95% CI)**	272.9 (234.4, 315.9)	287.2 (247.7, 331.2)	349.2 (305.4, 397.5)	346.5 (303.0, 394.5)	526.6 (472.5, 585.2)	512.6 (459.3, 570.3)

	Prespecified group of 12 cancer types[a]		All routinely staged cancer types excluding prostate cancer		All routinely staged cancer types	
	Intervention arm	Control arm	Intervention arm	Control arm	Intervention arm	Control arm
**Incidence rate difference (95% CI)**	−14.3 (−71.7, 43.0)		2.7 (−61.2, 66.6)		14.0 (−64.0, 92.1)	
**Incidence rate ratio (95% CI)**	0.95 (0.77, 1.17)		1.01 (0.84, 1.22)		1.03 (0.88, 1.20)	
**Third (incident) screening round**						
**Participants (n)**	62,867	63,101	62,867	63,101	62,867	63,101
**Participants with stage III/IV cancer, n (%)**	214 (0.34)	243 (0.39)	280 (0.45)	298 (0.47)	406 (0.65)	444 (0.70)

	Prespecified group of 12 cancer types[a]		All routinely staged cancer types excluding prostate cancer		All routinely staged cancer types	
	Intervention arm	Control arm	Intervention arm	Control arm	Intervention arm	Control arm
**Follow-up, person-years**	84,027	84,390	83,984	84,355	83,896	84,256
**Incidence rate per 100,000 person-years (95% CI)**	254.7 (221.7, 291.2)	287.9 (252.9, 326.5)	333.4 (295.5, 374.8)	353.3 (314.3, 395.7)	483.9 (438.0, 533.4)	527.0 (479.1, 578.3)
**Incidence rate difference (95% CI)**	−33.3 (−83.2, 16.5)		−19.9 (−76.0, 36.2)		−43.0 (−111.1, 25.0)	

	Prespecified group of 12 cancer types[a]		All routinely staged cancer types excluding prostate cancer		All routinely staged cancer types	
	Intervention arm	Control arm	Intervention arm	Control arm	Intervention arm	Control arm
**Incidence rate ratio (95% CI)**	0.88 (0.73, 1.07)		0.94 (0.80, 1.11)		0.92 (0.80, 1.05)	

[a]Lung, head and neck, colon/rectum, pancreas, myeloma/plasma cell neoplasm, liver/bile duct, stomach, esophagus, anus, lymphoma, ovary, and bladder.

Note: all results for the first and second screening rounds presented in this table are from post-hoc analyses.

Table S9. Reduction in stage IV cancers after each annual screening round in the NHS-Galleri trial.

	Prespecified group of 12 cancer types[a]		All routinely staged cancer types excluding prostate cancer		All routinely staged cancer types	
	**Intervention arm**	**Control arm**	**Intervention arm**	**Control arm**	**Intervention arm**	**Control arm**
**First (prevalent) screening round**						
**Participants (n)**	71,122	71,128	71,122	71,128	71,122	71,128
**Participants with stage IV cancer, n (%)**	117 (0.16)	128 (0.18)	137 (0.19)	150 (0.21)	164 (0.23)	169 (0.24)
**Follow-up, person-years**	70,254	70,259	70,244	70,251	70,227	70,242

	Prespecified group of 12 cancer types[a]		All routinely staged cancer types excluding prostate cancer		All routinely staged cancer types	
	Intervention arm	Control arm	Intervention arm	Control arm	Intervention arm	Control arm
**Incidence rate per 100,000 person-years (95% CI)**	166.5 (137.7, 199.6)	182.2 (152.0, 216.6)	195.0 (163.7, 230.6)	213.5 (180.7, 250.6)	233.5 (199.2, 272.1)	240.6 (205.7, 279.7)
**Incidence rate difference (95% CI)**	−15.6 (−59.5, 28.1)		−18.5 (−66.0, 28.9)		−7.1 (−58.1, 44.0)	
**Incidence rate ratio (95% CI)**	0.91 (0.71, 1.18)		0.91 (0.72, 1.16)		0.97 (0.78, 1.21)	

	Prespecified group of 12 cancer types[a]		All routinely staged cancer types excluding prostate cancer		All routinely staged cancer types	
	Intervention arm	Control arm	Intervention arm	Control arm	Intervention arm	Control arm
**Second (incident) screening round**						
**Participants (n)**	64,888	65,066	64,888	65,066	64,888	65,066
**Participants with Stage IV cancer, n (%)**	78 (0.12)	100 (0.15)	95 (0.15)	116 (0.18)	120 (0.18)	154 (0.24)
**Follow-up, person-years**	65,658	65,857	65,650	65,852	65,634	65,833

	Prespecified group of 12 cancer types[a]		All routinely staged cancer types excluding prostate cancer		All routinely staged cancer types	
	Intervention arm	Control arm	Intervention arm	Control arm	Intervention arm	Control arm
**Incidence rate per 100,000 person-years (95% CI)**	118.8 (93.9, 148.3)	151.8 (123.5, 184.7)	144.7 (117.1, 176.9)	176.2 (145.6, 211.3)	182.8 (151.6, 218.6)	233.9 (198.4, 273.9)
**Incidence rate difference (95% CI)**	−33.0 (−73.2, 6.7)		−31.4 (−75.1, 11.9)		−51.1 (−100.8, −1.8)	
**Incidence rate ratio (95% CI)**	0.78 (0.57, 1.06)		0.82 (0.62, 1.09)		0.78 (0.61, 1.00)	
**Third (incident) screening round**						

	Prespecified group of 12 cancer types[a]		All routinely staged cancer types excluding prostate cancer		All routinely staged cancer types	
	Intervention arm	Control arm	Intervention arm	Control arm	Intervention arm	Control arm
**Participants (n)**	62,867	63,101	62,867	63,101	62,867	63,101
**Participants with stage IV cancer, n (%)**	104 (0.17)	142 (0.23)	130 (0.21)	165 (0.26)	158 (0.25)	198 (0.31)
**Follow-up, person-years**	84,118	84,457	84,102	84,445	84,081	84,423
**Incidence rate per 100,000 person-years (95% CI)**	123.6 (101.0, 149.8)	168.1 (141.6, 198.2)	154.6 (129.1, 183.5)	195.4 (166.7, 227.6)	187.9 (159.8, 219.6)	234.5 (203.0, 269.6)

	Prespecified group of 12 cancer types[a]		All routinely staged cancer types excluding prostate cancer		All routinely staged cancer types	
	Intervention arm	Control arm	Intervention arm	Control arm	Intervention arm	Control arm
Incidence rate difference (95% CI)	−44.5 (−81.3, −8.1)		−40.8 (−81.0, −0.9)		−46.6 (−90.7, −2.8)	
Incidence rate ratio (95% CI)	0.74 (0.57, 0.95)		0.79 (0.62, 1.00)		0.80 (0.65, 0.99)	

[a]Lung, head and neck, colon/rectum, pancreas, myeloma/plasma cell neoplasm, liver/bile duct, stomach, esophagus, anus, lymphoma, ovary, and bladder.

Table S10. Reduction in advanced cancers after 3 screening rounds.

	Prespecified group of 12 cancer types[a]		All routinely staged cancer types excluding prostate cancer		All routinely staged cancer types	
	Intervention arm (N = 71,122)	Control arm (N = 71,128)	Intervention arm (N = 71,122)	Control arm (N = 71,128)	Intervention arm (N = 71,122)	Control arm (N = 71,128)
**Participants with advanced cancer, n (%)**	771 (1.08%)	751 (1.06%)	1052 (1.48%)	998 (1.40%)	1415 (1.99%)	1359 (1.91%)
**Follow-up, person-years**	234,888	235,181	234,522	234,916	233,912	234,362

	Prespecified group of 12 cancer types[a]		All routinely staged cancer types excluding prostate cancer		All routinely staged cancer types	
	**Intervention arm (N = 71,122)**	**Control arm (N = 71,128)**	**Intervention arm (N = 71,122)**	**Control arm (N = 71,128)**	**Intervention arm (N = 71,122)**	**Control arm (N = 71,128)**
**Incidence rate per 100,000 person-years (95% CI)**	328.2 (305.5, 352.3)	319.3 (296.9, 343.0)	448.6 (421.9, 476.5)	424.8 (398.9, 452.0)	604.9 (573.8, 637.3)	579.9 (549.4, 611.5)
**Incidence rate difference (95% CI)**	8.9 (-23.6, 41.5)		23.7 (-14.1, 61.6)		25.1 (-19.0, 69.2)	
**Incidence rate ratio (95% CI)**	1.03 (0.93, 1.14)		1.06 (0.97, 1.15)		1.04 (0.97, 1.12)	

[a]Lung, head and neck, colon/rectum, pancreas, myeloma/plasma cell neoplasm, liver/bile duct, stomach, esophagus, anus, lymphoma, ovary, and bladder.

Note: Incidence rates and corresponding 95% CIs intervals for each arm were calculated using exact Poisson (Garwood) methods. The 95% CI for the incidence rate difference was derived using the Method of Variance Estimates Recovery with Jeffreys equal-tailed single-rate intervals (MOVER-J; Laud, 2017). The Jeffreys-based MOVER approach was selected for the between-arm comparison because the direct combination of 2 Garwood intervals does not guarantee nominal coverage for a difference, whereas MOVER-J maintains near-exact coverage and balanced precision.

Table S11. Reduction in stage III/IV cancers, excluding breast, cervix and colon/rectum cancers after 3 screening rounds.

	Intervention arm (N = 71,122)	Control arm (N = 71,128)
**Participants with stage III/IV cancer, n (%)**	1077 (1.51%)	1030 (1.45%)
**Follow-up, person-years**	234,387	234,762
**Incidence rate per 100,000 person-years (95% CI)**	459.5 (432.5, 487.8)	438.7 (412.4, 466.4)
**Incidence rate difference (95% CI)**	20.8 (-17.6, 59.1)	
**Incidence rate ratio (95% CI)**	1.05 (0.96, 1.14)	

Note: Incidence rates and corresponding 95% CIs for each arm were calculated using exact Poisson (Garwood) methods. The 95% CI for the incidence rate difference was derived using the Method of Variance Estimates Recovery with Jeffreys equal-tailed single-rate intervals (MOVER-J; Laud, 2017). The Jeffreys-based MOVER approach was selected for the between-arm comparison because the direct combination of 2 Garwood intervals does not guarantee nominal coverage for a difference, whereas MOVER-J maintains near-exact coverage and balanced precision.

Table S12. Cancer detection rates in the first (prevalent) screening round.

	Intervention arm (N = 71,122)	Control arm (N = 71,128)
**Cancer participants**	1145	950
**Cancer detection rate, % (95% CI)**	1.61 (1.52, 1.71)	1.34 (1.25, 1.42)
**Cancer detection rate ratio (95% CI)[a]**	1.21 (1.11, 1.31)	

[a]95% CI for ratios of proportions were calculated using the Wald method on the log-transformed ratio.

Table S13. Proportion of cancers by stage in the third (incident) screening round.

	Intervention arm (N = 71,122)	Control arm (N = 71,128)
**Participants with routinely staged cancer**[a]	1208	1182
**Stage I, n (%)**	516 (42.7)	487 (41.2)
**Stage II, n (%)**	243 (20.1)	221 (18.7)
**Stage III, n (%)**	248 (20.5)	246 (20.8)
**Stage IV, n (%)**	158 (13.1)	198 (16.8)
**Missing, n (%)**[b]	43 (3.6)	30 (2.5)
**Stage I/II, n (%)**	759 (62.8)	708 (59.9)
**Difference in stage I/II proportion (95% CI)**[c]	2.93 (-0.97, 6.82)	

[a]Cancer stage per the highest routinely staged cancer.

[b]Missing includes those cancers with insufficient information/not staged.

[c]95% CI for difference in proportions calculated using Newcombe's method based on Wilson score intervals.

Table S14. Proportion of cancers by stage after 3 screening rounds in the NHS-Galleri trial.

	Prespecified group of 12 cancer types[a]		All routinely staged cancer types, excluding prostate cancer		All routinely staged cancer types	
	Intervention arm (N = 71,122)	Control arm (N = 71,128)	Intervention arm (N = 71,122)	Control arm (N = 71,128)	Intervention arm (N = 71,122)	Control arm (N = 71,128)
**Cancer participants[b]**	1421	1298	2431	2228	3431	3177
**Stage I, n (%)**	421 (29.6)	370 (28.5)	938 (38.6)	897 (40.3)	1383 (40.3)	1308 (41.2)
**Stage II, n (%)**	226 (15.9)	189 (14.6)	494 (20.3)	415 (18.6)	673 (19.6)	574 (18.1)
**Stage III, n (%)**	360 (25.3)	287 (22.1)	489 (20.1)	376 (16.9)	763 (22.2)	645 (20.3)
**Stage IV, n (%)**	341 (24.0)	394 (30.4)	410 (16.9)	464 (20.8)	503 (14.7)	563 (17.7)
**Missing[c], n (%)**	73 (5.1)	58 (4.5)	98 (4.0)	74 (3.3)	107 (3.1)	85 (2.7)
**Stage I/II, n (%)**	647 (45.5)	559 (43.1)	1432 (58.9)	1312 (58.9)	2056 (59.9)	1882 (59.2)

	Prespecified group of 12 cancer types[a]		All routinely staged cancer types, excluding prostate cancer		All routinely staged cancer types	
	Intervention arm (N = 71,122)	Control arm (N = 71,128)	Intervention arm (N = 71,122)	Control arm (N = 71,128)	Intervention arm (N = 71,122)	Control arm (N = 71,128)
**Relative risk in stage I/II (95% CI)[d]**	1.16 (1.03, 1.30)		1.09 (1.01, 1.18)		1.09 (1.03, 1.16)	
**Stage I/II/III, n (%)**	1007 (70.9)	846 (65.2)	1921 (79.0)	1688 (75.8)	2819 (82.2)	2527 (79.5)
**Relative risk in stage I/II/III (95% CI)[d]**	1.19 (1.09, 1.30)		1.14 (1.07, 1.21)		1.12 (1.06, 1.18)	

[a]Lung, head and neck, colon/rectum, pancreas, myeloma/plasma cell neoplasm, liver/bile duct, stomach, esophagus, anus, lymphoma, ovary, and bladder.

[b]Stage 0 cancers are not presented in the table, but are included in the total number of cancer participants.

[c]Missing includes those cancers with insufficient information/not staged.

[d]Relative risks and 95% confidence intervals were obtained using the Wald method on the log scale under binomial sampling assumptions.

Note: all results presented in this table are from a post-hoc analysis.

Table S15. Program sensitivity for MCED and NHS Screening.

	Overall		First screening round		Second screening round		Third screening round	
	Intervention arm (N = 71,122)	Control arm (N = 71,128)	Intervention arm (N = 71,122)	Control arm (N = 71,128)	Intervention arm (N = 71,122)	Control arm (N = 71,128)	Intervention arm (N = 71,122)	Control arm (N = 71,128)
**Screen-detected cancer, n**	1173	290	494	92	318	70	346	110
**Program sensitivity for screening, % (95% CI)**	32.25 (30.75, 33.79)	8.53 (7.64, 9.52)	43.14 (40.30, 46.03)	9.68 (7.96, 11.73)	32.19 (29.35, 35.16)	7.17 (5.72, 8.96)	26.95 (24.59, 29.44)	8.68 (7.25, 10.36)

Note: all results for "program sensitivity for screening" presented in this table are from post-hoc analyses.

Table S16. Safety results by arm: number of participants with related adverse events (AEs) across all 3 screening rounds.

Number of participants with at least one related AE	Intervention arm (N = 71,122)			Control arm (N = 71,128)			Not randomized (N = 576)
	On blood draw visit, n (%)	Off blood draw visit, n (%)	Overall, n (%)	On blood draw visit, n (%)	Off blood draw visit, n (%)	Overall, n (%)	Overall, n (%)
**Total**	258 (0.36)	115 (0.16)	371 (0.52)	312 (0.44)	13 (0.02)	321 (0.45)	30 (5.21)
**AE relatedness**							
Study procedure related[a]	258 (0.36)	80 (0.11)	337 (0.47)	312 (0.44)	13 (0.02)	321 (0.45)	30 (5.21)
Phlebotomy	250 (0.35)	17 (0.02)	266 (0.37)	302 (0.43)	12 (0.02)	310 (0.44)	30 (5.21)
Other	10 (0.01)	63 (0.09)	73 (0.10)	12 (0.02)	1 (<0.01)	13 (0.02)	0 (0)
Device related	0 (0)	27 (0.04)	27 (0.04)	0 (0)	0 (0)	0 (0)	0 (0)
Study procedure related and device related	0 (0)	11 (0.02)	11 (0.02)	0 (0)	0 (0)	0 (0)	0 (0)

Number of participants with at least one related AE	Intervention arm (N = 71,122)			Control arm (N = 71,128)			Not randomized (N = 576)
	On blood draw visit, n (%)	Off blood draw visit, n (%)	Overall, n (%)	On blood draw visit, n (%)	Off blood draw visit, n (%)	Overall, n (%)	Overall, n (%)
**AE Severity**							
Mild	222 (0.31)	81 (0.11)	301 (0.42)	272 (0.38)	9 (0.01)	277 (0.39)	23 (3.99)
Moderate	37 (0.05)	34 (0.05)	71 (0.10)	39 (0.06)	4 (0.01)	43 (0.06)	8 (1.39)
Severe	2 (<0.01)	2 (<0.01)	4 (0.01)	2 (<0.01)	1 (<0.01)	3 (<0.01)	0 (0)
**AE Outcome**							
Recovering / resolving	51 (0.07)	13 (0.02)	64 (0.09)	61 (0.09)	3 (<0.01)	64 (0.09)	1 (0.17)
Recovered / resolved	202 (0.28)	45 (0.06)	247 (0.35)	249 (0.35)	7 (0.01)	255 (0.36)	29 (5.04)
Recovered / resolved with sequelae	1 (<0.01)	0 (0)	1 (<0.01)	0 (0.00)	0 (0)	0 (0)	0 (0)

Number of participants with at least one related AE	Intervention arm (N = 71,122)			Control arm (N = 71,128)			Not randomized (N = 576)
	On blood draw visit, n (%)	Off blood draw visit, n (%)	Overall, n (%)	On blood draw visit, n (%)	Off blood draw visit, n (%)	Overall, n (%)	Overall, n (%)
Not recovered / not resolved	4 (0.01)	31 (0.04)	35 (0.05)	4 (0.01)	3 (<0.01)	7 (0.01)	1 (0.17%)
Fatal	0 (0)	0 (0)	0 (0)	0 (0)	0 (0)	0 (0)	0 (0)
Unknown	2 (<0.01)	33 (0.05)	35 (0.05)	0 (0)	1 (<0.01)	1 (<0.01)	0 (0)
**Serious AE**	0 (0)	0 (0)	0 (0)	0 (0)	0 (0)	0 (0)	0 (0)
**Unexpected AE**	10 (0.01)	39 (0.06)	49 (0.07)	8 (0.01)	6 (0.01)	14 (0.02)	1 (0.17)
**Serious and unexpected AE**	0 (0)	0 (0)	0 (0)	0 (0)	0 (0)	0 (0)	0 (0)

[a]After all 3 screening rounds, 1 event has been reported with multiple criteria (phlebotomy and other).

Note: the results in the “total” row presented in this table are from a post-hoc analysis.

Table S17. Safety results by arm: number of related adverse events (AEs) across all 3 screening rounds.

Number of related AEs	Intervention arm (N = 71,122)			Control arm (N = 71,128)			Not randomized (N = 576)
	On blood draw visit	Off blood draw visit	Overall	On blood draw visit	Off blood draw visit	Overall	Overall
**Total**	267	123	390	324	14	338	33
**AE relatedness**							
Study procedure related	267	84	351	324	14	338	33
Phlebotomy	258	18	276	312	13	325	33
Other	9	66	75	12	1	13	0
Device related	0	28	28	0	0	0	0
Study procedure related and device related	0	11	11	0	0	0	0
**AE Severity**							

Number of related AEs	Intervention arm (N = 71,122)			Control arm (N = 71,128)			Not randomized (N = 576)
	On blood draw visit	Off blood draw visit	Overall	On blood draw visit	Off blood draw visit	Overall	Overall
Mild	228	85	313	282	9	291	23
Moderate	37	36	73	40	4	44	10
Severe	2	2	4	2	1	3	0
**AE Outcome**							
Recovering / resolving	51	13	64	61	3	64	1
Recovered / resolved	209	45	254	259	7	266	31
Recovered / resolved with sequelae	1	0	1	0	0	0	0
Not recovered / not resolved	4	32	36	4	3	7	1

Number of related AEs	Intervention arm (N = 71,122)			Control arm (N = 71,128)			Not randomized (N = 576)
	On blood draw visit	Off blood draw visit	Overall	On blood draw visit	Off blood draw visit	Overall	Overall
Fatal	0	0	0	0	0	0	0
Unknown	2	33	35	0	1	1	0
**Serious AE**	0	0	0	0	0	0	0
**Unexpected AE**	10	41	51	8	6	14	3
**Serious and unexpected AE**	0	0	0	0	0	0	0

Note: the results in the “total” row presented in this table are from a post-hoc analysis.

Table S18. Self-reported adherence to standard-of-care cancer screening.

	First screening round		Second screening round		Third screening round	
	Intervention arm (N = 71,122)	Control arm (N = 71,128)	Intervention arm (N = 64,888)	Control arm (N = 65,066)	Intervention arm (N = 62,867)	Control arm (N = 63,101)
I have never heard of cancer screening, n (%)[a]	2056 (2.9)	2108 (3.0)	937 (1.5)	971 (1.6)	561 (0.9)	582 (0.9)
I have heard of cancer screening but have never been invited, n (%)[a]	13,955 (19.6)	13,871 (19.5)	5110 (8.2)	5137 (8.2)	3273 (5.2)	3294 (5.2)
I have been invited for cancer screening but have never done it, n (%)[a]	861 (1.2)	883 (1.2)	575 (0.9)	551 (0.9)	519 (0.8)	529 (0.8)
I have been invited for cancer screening but have not done it every time I was invited, n (%)[a]	2428 (3.4)	2361 (3.3)	1923 (3.1)	1876 (3.0)	1625 (2.6)	1584 (2.5)

	First screening round		Second screening round		Third screening round	
	Intervention arm (N = 71,122)	Control arm (N = 71,128)	Intervention arm (N = 64,888)	Control arm (N = 65,066)	Intervention arm (N = 62,867)	Control arm (N = 63,101)
**I have been invited for cancer screening and have done it every time I was invited, n (%)[a]**	51,013 (71.8)	51,150 (72.0)	53,433 (86.0)	53,619 (86.1)	56,711 (90.2)	56,890 (90.2)
**I don't know, n (%)[a]**	655 (0.9)	608 (0.9)	133 (0.2)	141 (0.2)	156 (0.2)	185 (0.3)
**I decline to answer this question, n (%)[a]**	51 (0.1)	58 (0.1)	18 (<0.1)	15 (<0.1)	17 (<0.1)	27 (<0.1)
**Non-missing, n**	71,019	71,039	62,129	62,310	62,862	63,091
**Missing, n**	103	89	2759	2756	5	10
**Response rate[b] (%)**	99.9	99.9	95.7	95.8	100	100

[a]Percentage is based on the count out of all non-missing responses.

[b]Response rate in the second screening round is based on non-missing responses out of all participants who have had a second round blood draw. Originally, only the change of attitude question was to be asked at the second screening round. About 1 month into the

second screening round, the protocol was changed so that participants in the second screening round would also be asked to complete this questionnaire. Participants attending their second blood draw during the first month of the second screening round roll-out were therefore never asked to complete this questionnaire, hence a larger percentage missing compared to the first screening round.

References

1. Swanton C, Bachtiar V, Mathews C, et al. NHS-Galleri trial: enriched enrolment approaches and sociodemographic characteristics of enrolled participants. Clin Trials 2025;22(2):227–38.

2. U.S. Food & Drug Administration. Investigator responsibilities – safety reporting for investigational drugs and devices [Internet]. 2025 [cited 2026 June 30];Available from: https://www.fda.gov/regulatory-information/search-fda-guidance-documents/investigator-responsibilities-safety-reporting-investigational-drugs-and-devices

3. Lowenhoff I, Dolly S, Dowinton Smith R, et al. Clinical referral to the NHS following multi-cancer early detection test results from the NHS-Galleri trial. Front Oncol 2025;15:1511816.

4. Neal RD, Johnson P, Clarke CA, et al. Cell-free DNA–based multi-cancer early detection test in an asymptomatic screening population (NHS-Galleri): design of a pragmatic, prospective randomised controlled trial. Cancers 2022;14(19):4818.
